# Conformal On-Body Antenna System Integrated with Deep Learning for Non-Invasive Breast Cancer Detection

**DOI:** 10.3390/s25154670

**Published:** 2025-07-28

**Authors:** Marwa H. Sharaf, Manuel Arrebola, Khalid F. A. Hussein, Asmaa E. Farahat, Álvaro F. Vaquero

**Affiliations:** 1 Electronics and Communications Department, College of Engineering and Technology, Arab Academy for Science, Technology & Maritime Transport, Alexandria 21937, Egypt; 2 Universidad de Oviedo, 33007 Gijón, Spain; 3 Information Processing and Telecommunications Center, Universidad Politécnica de Madrid, 28040 Madrid, Spain; manuel.arrebola@upm.es; 4 Microwave Engineering Department, Electronics Research Institute (ERI), Cairo 11843, Egypt; fkhalid@eri.sci.eg (K.F.A.H.); e_asma_e@yahoo.com (A.E.F.); 5 Group of Signal Theory and Communications, Department of Mathematics, Universidad de Oviedo, 33007 Gijón, Spain

**Keywords:** microwave imaging, breast cancer detection, antenna design, deep learning, neural networks, antenna arrays, specific absorption rate (SAR), dielectric properties, microwave radar system, ultra-wideband antenna

## Abstract

Breast cancer detection through non-invasive and accurate techniques remains a critical challenge in medical diagnostics. This study introduces a deep learning-based framework that leverages a microwave radar system equipped with an arc-shaped array of six antennas to estimate key tumor parameters, including position, size, and depth. This research begins with the evolutionary design of an ultra-wideband octagram ring patch antenna optimized for enhanced tumor detection sensitivity in directional near-field coupling scenarios. The antenna is fabricated and experimentally evaluated, with its performance validated through S-parameter measurements, far-field radiation characterization, and efficiency analysis to ensure effective signal propagation and interaction with breast tissue. Specific Absorption Rate (SAR) distributions within breast tissues are comprehensively assessed, and power adjustment strategies are implemented to comply with electromagnetic exposure safety limits. The dataset for the deep learning model comprises simulated self and mutual S-parameters capturing tumor-induced variations over a broad frequency spectrum. A core innovation of this work is the development of the Attention-Based Feature Separation (ABFS) model, which dynamically identifies optimal frequency sub-bands and disentangles discriminative features tailored to each tumor parameter. A multi-branch neural network processes these features to achieve precise tumor localization and size estimation. Compared to conventional attention mechanisms, the proposed ABFS architecture demonstrates superior prediction accuracy and interpretability. The proposed approach achieves high estimation accuracy and computational efficiency in simulation studies, underscoring the promise of integrating deep learning with conformal microwave imaging for safe, effective, and non-invasive breast cancer detection.

## 1. Introduction

Breast cancer remains one of the leading causes of mortality among women worldwide, highlighting the urgent need for non-invasive, cost-effective, and early detection techniques [1]. Conventional imaging modalities such as mammography, diagnostic ultrasound, and MRI, though widely used, face limitations including exposure to ionizing radiation, high operational costs, and reduced effectiveness in women with dense breast tissue [2,3]. Microwave imaging has emerged as a promising alternative due to its non-ionizing nature, low cost, and ability to detect malignant tumors based on dielectric contrast between healthy and cancerous tissues [4].

Accurate localization and characterization of breast tumors using microwave techniques, however, remains challenging due to complex tissue heterogeneity and multipath electromagnetic interactions [5,6,7]. Traditional approaches relying on delay-and-sum beamforming or inverse scattering often suffer from limited resolution and sensitivity to noise and model mismatch [8,9].

To address these limitations, deep learning has been increasingly explored for its ability to learn nonlinear mappings from high-dimensional electromagnetic data to tumor-related features [6,10,11]. Recent reviews and studies have comprehensively highlighted the potential of deep learning for microwave breast imaging, demonstrating improvements in detection accuracy and robustness compared to classical reconstruction techniques [3,12,13,14].

In recent years, deep learning has played a transformative role in biomedical image analysis, with substantial advances demonstrated in segmentation and classification challenges such as MoNuSAC2020 [15]. This benchmark addressed the segmentation and classification of nuclei across multiple organs using histopathology images, illustrating the power of convolutional architectures and data-driven approaches in extracting fine-grained diagnostic features. Unlike such optical imaging modalities, our work addresses non-invasive tumor detection using microwave-based data, where direct spatial imaging is not feasible and learning must be inferred from S-parameter signatures. This introduces unique challenges in data representation, interpretation, and model design, thereby motivating the customized multi-branch architecture proposed in this study.

This work proposes a novel system that integrates a conformal, skin-mounted antenna array with a deep learning-based tumor detection framework. A key contribution is the introduction of an attention-based frequency selection (ABFS) mechanism within the model, enabling it to learn frequency-specific features most relevant to tumor location and size. By prioritizing informative sub-bands, the ABFS model improves generalization and enhances tumor localization accuracy compared to fixed-band feature extraction methods [16,17,18].

The proposed system uses mutual and self S-parameters captured by the conformal antenna array placed directly on the breast surface. These electromagnetic signatures are mapped to tumor parameters such as spatial location and dimensions using a custom multi-branch neural network architecture, where each branch estimates specific tumor attributes using distinct frequency sub-band features [19,20] 

To validate the system, we fabricate and test the flexible antenna and simulate a breast model with layered tissues and embedded tumors. The antenna’s performance is evaluated in both flat and curved configurations, ensuring real-world adaptability. Specific Absorption Rate (SAR) analysis is also performed to confirm safety compliance for human exposure [21,22].

This paper demonstrates that the combination of conformal microwave hardware with data-driven intelligence enables a compact, low-cost, and accurate approach for non-invasive breast cancer detection. The rest of the paper is organized as follows:

Section 2 presents the antenna design and evaluation, including the parametric study. Section 3 discusses the breast model. Section 4 describes the SAR distribution in the curved model of the breast. Section 5 details the antenna measurements. Section 6 discusses the off-body antenna performance. Section 7 provides comparative antenna performance analysis. Section 8 describes the reduced antenna system and the truncated breast model developed to enable a more efficient deep learning algorithm. Section 9 and Section 10 detail the dataset, deep learning model, and tumor detection results. Section 11 discusses the limitations of the present work and outlines directions for future extensions. Finally, Section 12 concludes the paper.

## 2. Antenna Design

The antenna is designed as an annular patch with an eight-pointed star shape, fed through a probe. It is intended to operate with the radiating patch in direct contact with the skin, while the ground plane faces free space. This configuration ensures that the majority of the electromagnetic energy is directed into the breast tissues while minimizing radiation into free space. A three-layer human tissue model (skin, fatty mammary glands, and muscle) is used to facilitate simulation.

### 2.1. Geometry of the Antenna

**Figure 1:**
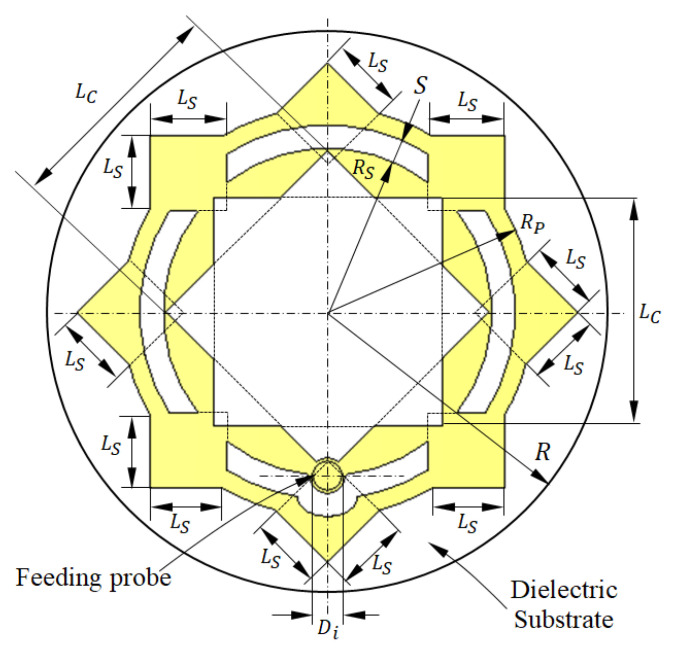
Design and dimensional parameters of the radiating patch of the antenna proposed for breast cancer detection.

The proposed patch antenna can be regarded as composed of a circular patch surrounded by an external ring. The circular patch has a hole shaped like an eight-pointed star. The perforated circular patch and the ring are interconnected by metallic square segments that are uniformly distributed over the circumference, as shown in Figure 1. The patch is printed on a circular substrate of radius R, height h, dielectric constant $\varepsilon_{r}$, and loss tangent tanδ. The substrate is backed by a circular ground plane of the same radius as the substrate.

**Figure 2:**
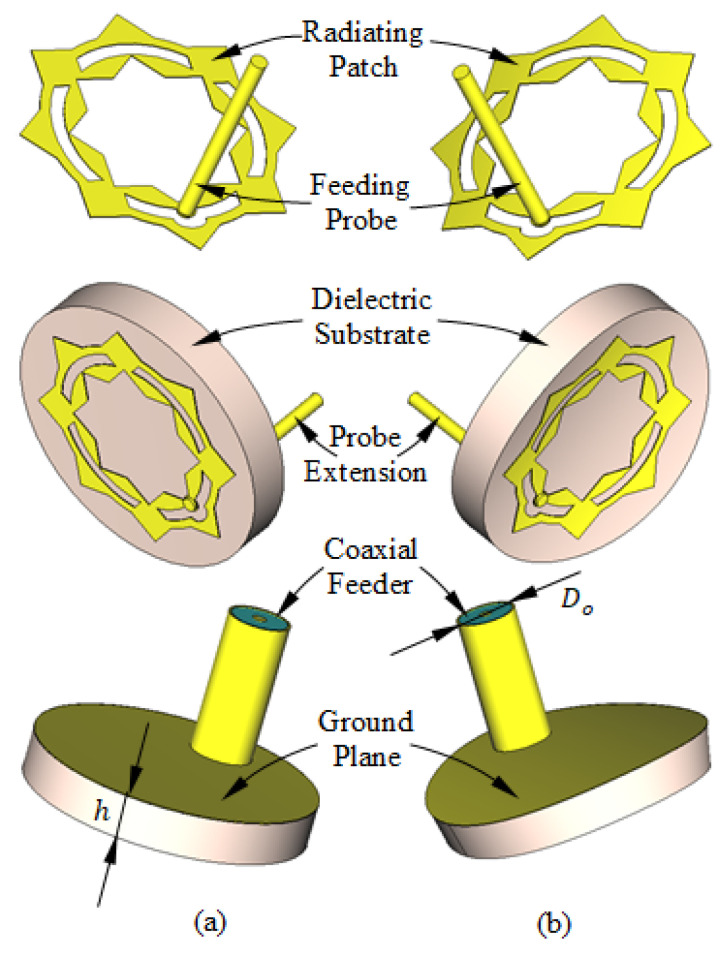
Structure of the antenna proposed for breast cancer detection. (**a**) Flat antenna. (**b**) Curved antenna.

Three-dimensional views of the different parts of the proposed antenna are presented in Figure 2. To be conformal to the surface of the human breast, this antenna uses a flexible substrate of the material Rogers’ RO3003™ (Rogers corporations, Lancashire, UK). This antenna can be either flat or curved with a given bend radius to fit the curvature of the breast according to the location at which it is mounted. The antenna is fed through a 50 Ω coaxial line whose outer conductor is welded to the circular ground backing the substrate. The probe feeding the patch is formed by extending the inner conductor of the coaxial line to penetrate through the substrate and to be welded to the radiating patch at the location indicated in Figure 1.

The optimum dimensions of the antenna shown in Figure 1 and Figure 2 are given in Table 1. These values are obtained through a complete parametric study using the CST simulator to obtain the maximum bandwidth of impedance matching. Some examples of the performed parametric study are presented in Section 2.2 for both the flat and curved antenna structures.

### 2.2. Antenna Design Evolution and Optimization

The proposed antenna was developed through a sequence of progressive design refinements and parametric optimization to achieve a wide impedance matching bandwidth suitable for breast cancer detection.

#### 2.2.1. Design Stages

**Figure 3:**
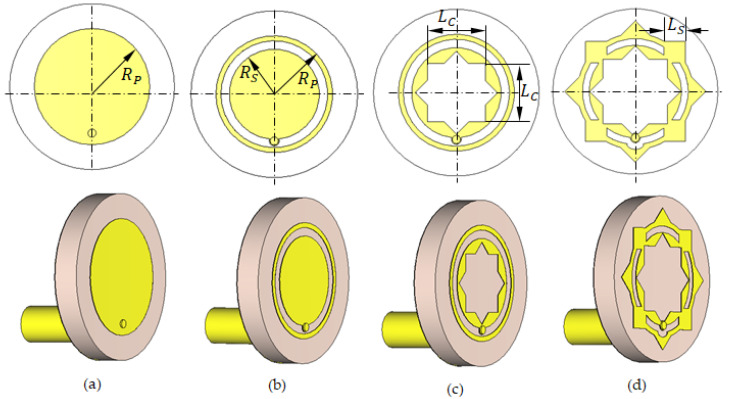
Geometries of the progressive design stages of the proposed antenna: (**a**) Stage 1. (**b**) Stage 2. (**c**) Stage 3. (**d**) Stage 4 (Final).

**Figure 4:**
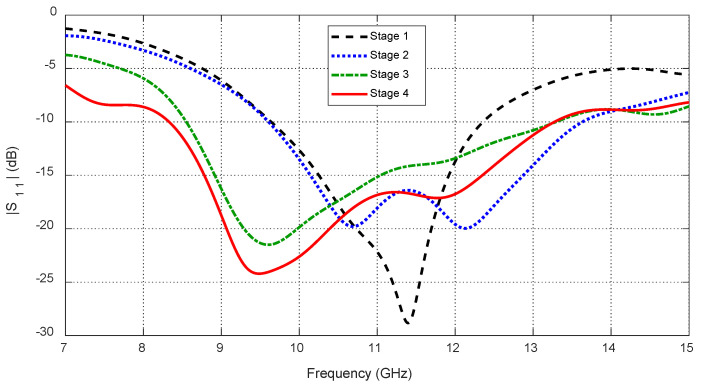
Continuous improvement of the frequency response of the reflection coefficient with the progressive design stages of the proposed antenna (see Figure 3).

The initial concept started with a circular patch on a circular substrate (Stage 1, Figure 3a), achieving impedance matching between 9.6 and 12.3 GHz (Figure 4). To extend the bandwidth, an annular slot of width S and radius $R_{S}$ was introduced (Stage 2, Figure 3b), improving the impedance matching band to 9.6–13.7 GHz (Figure 4). In Stage 3 (Figure 3c), an eight-pointed star-shaped aperture, formed by two orthogonal square cuts, transformed the structure into concentric rings and shifted the lower frequency edge to 8.6 GHz (Figure 4). Finally, Stage 4 (Figure 3d) interconnected the rings with four square patches and added triangular protrusions to maintain symmetry, achieving an impedance bandwidth of 8.3–13.3 GHz (Figure 4).

#### 2.2.2. Parametric Optimization

**Figure 5:**
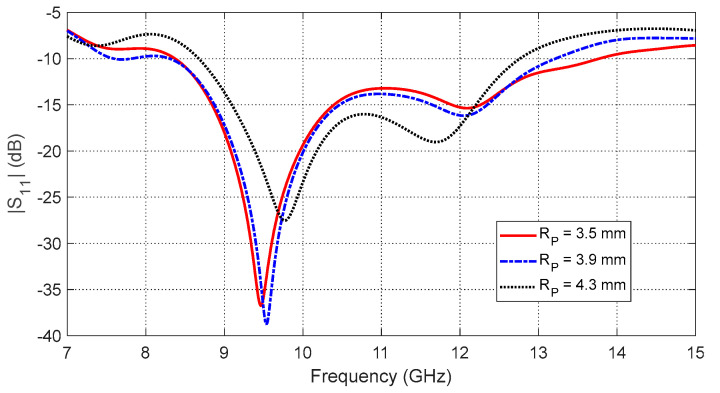
Frequency response of the reflection coefficient magnitude, $\left|S_{11}\right|$, of the curved antenna on the curved breast model for different values of the annular patch radius, $R_{P}$.

**Figure 6:**
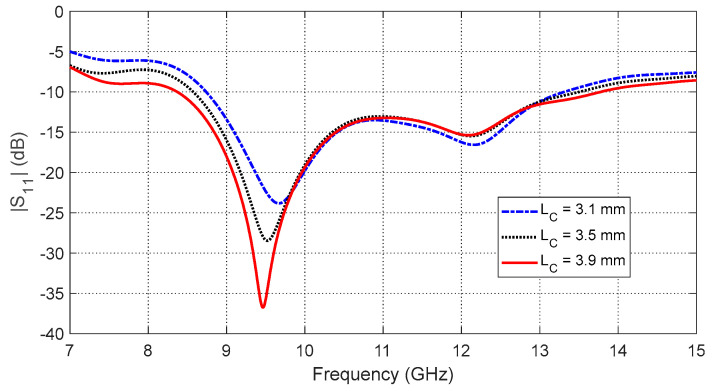
Frequency response of the reflection coefficient magnitude, $\left|S_{11}\right|$, of the curved antenna on the curved breast model for different values of the aperture side length, $L_{C}$.

**Figure 7:**
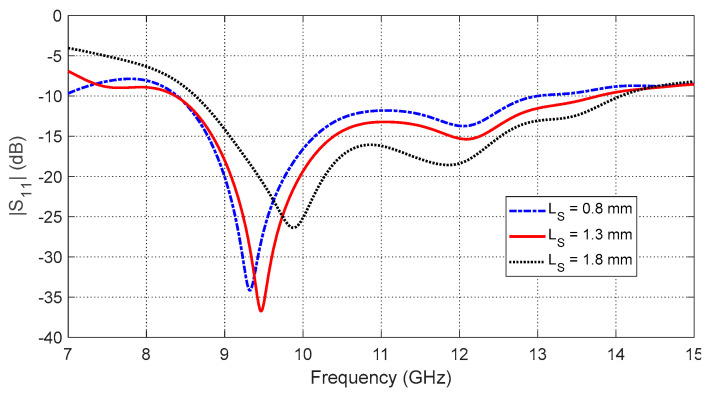
Frequency response of the reflection coefficient magnitude, $\left|S_{11}\right|$, for the curved antenna on the curved breast model. The response is shown for varying side lengths, $L_{S}$, of the square segments arranged along the perimeter of the annular patch.

A systematic parametric study was then performed to fine-tune the key geometric parameters and ensure robust performance on the breast model (Section 3). Using CST^®^ simulations, the following parameters were varied:

− The annular patch radius ($R_{P}$) affects the start and end frequencies of the impedance matching band as shown in Figure 5. The optimal value is $R_{P}=3.5\;mm$, yielding the widest frequency band.
− The aperture side length ($L_{C}$) determines the resonance behavior of the eight-pointed star aperture (Figure 6). The optimal dimension is $L_{C}=3.9\;mm$.
− The interconnection segment size ($L_{S}$) controls impedance matching across the band as shown in Figure 7. The best performance is obtained with $L_{S}=1.3\;mm$.

These optimized dimensions were adopted in the final antenna geometry, shown in Figure 1 and Figure 2, ensuring wideband performance across the target 8.3–13.3 GHz range and suitability for conformal mounting.

### 2.3. Innovation in Antenna Geometry

The proposed octagram ring patch antenna introduces several novel design features that distinguish it from existing flexible antennas developed for biomedical and wearable applications. While ring-based and star-shaped geometries have been reported in the literature, our octagram configuration is uniquely tailored for on-body, non-invasive breast cancer detection in the microwave frequency range. The key innovative aspects are highlighted in the following subsections

#### 2.3.1. High-Density Current Folding for Bandwidth Enhancement

The octagram contour creates sharply angled surface current paths, effectively increasing their traversal length without enlarging the antenna’s physical footprint. This geometric folding supports multiple resonant modes, thereby enhancing impedance bandwidth—an advantage that is particularly important for broadband tissue interrogation.

#### 2.3.2. Integrated Slot-Loading for Directional Near-Field Coupling

Unlike conventional designs that prioritize far-field gain, the proposed structure incorporates symmetric slot-loading within the octagram to produce a concentrated, shallow near-field distribution. This approach improves energy penetration into breast tissue and increases mutual coupling sensitivity across antenna pairs, both of which are critical for accurate S-parameter-based detection.

#### 2.3.3. On-Body Stability and Low-SAR Conformity

The octagram design, fabricated on a flexible polyimide substrate, maintains stable radiation characteristics under skin-conformal bending. Moreover, the antenna achieves a peak Specific Absorption Rate (SAR) well below IEEE C95.1 safety limits, ensuring safe operation while maximizing field interaction depth.

#### 2.3.4. Application-Driven Optimization

Unlike prior ring or star-shaped designs typically evaluated in free space or simplistic phantoms, our antenna has been optimized and validated on anatomically realistic hemispherical breast models with skin-equivalent dielectric properties. The inclusion of a back reflector layer and a matched feed structure further enhances in-body coupling efficiency.

Table 2 demonstrates that the proposed octagram ring antenna achieves a unique combination of compact size, wide operating bandwidth, and acceptable SAR levels compared to representative flexible antennas reported in the literature. Notably, while previous designs such as the circular ring [23] and star-shaped geometries [24] operate below 3.5 GHz with physical dimensions exceeding 40 mm on each side, our antenna achieves broadband operation over 8.3–13.3 GHz within an ultra-compact footprint of only 10×10×0.25 mm. This substantial reduction in size, combined with the tailored near-field coupling and impedance matching enhancements, underscores the novelty and suitability of the proposed design for non-invasive, on-body breast imaging applications. To our knowledge, this is the first flexible microwave antenna integrating an octagram contour with embedded slots specifically optimized for broadband tissue interrogation in wearable cancer detection systems.

## 3. Three-Layer Model of the Human Breast

**Figure 8:**
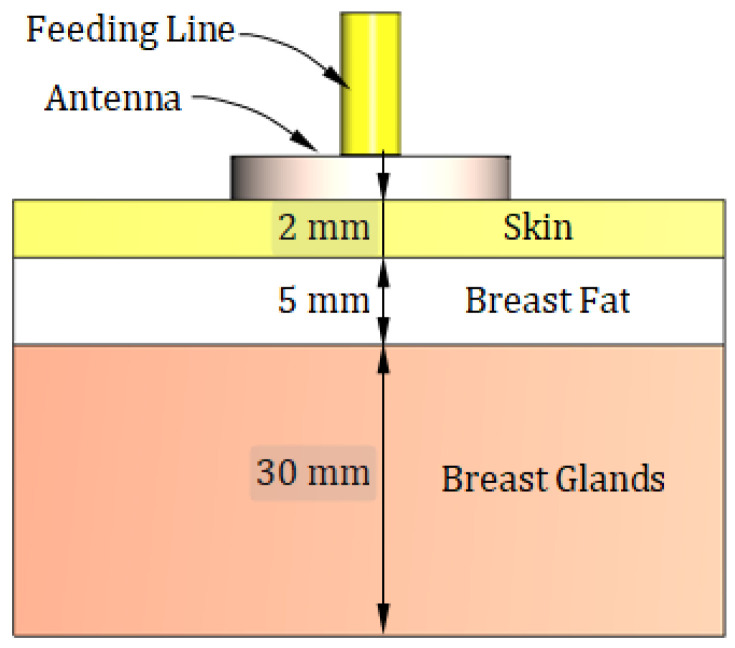
Sketched drawing of the flat model of a portion of the human breast employed for simulation of the proposed antenna.

**Figure 9:**
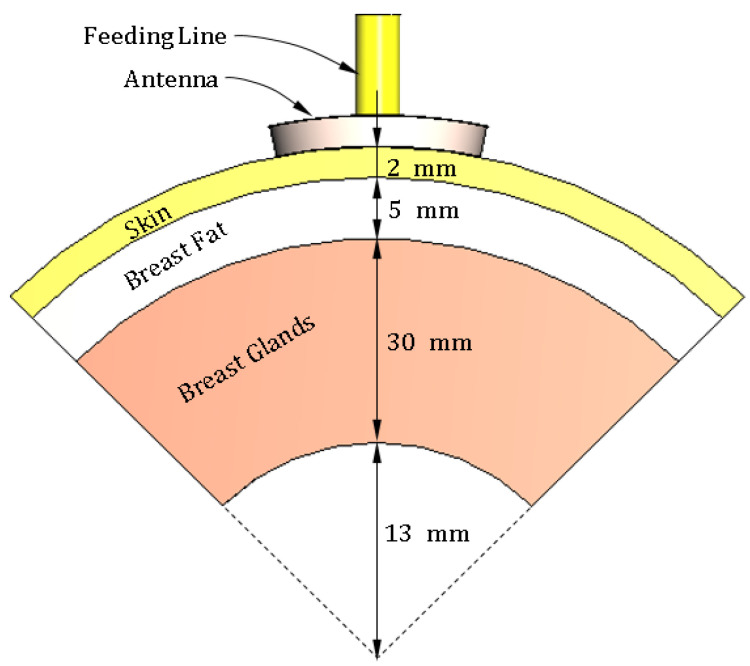
Sketched drawing of the curved model for a sector of the human breast employed for simulation of the proposed antenna.

**Figure 10:**
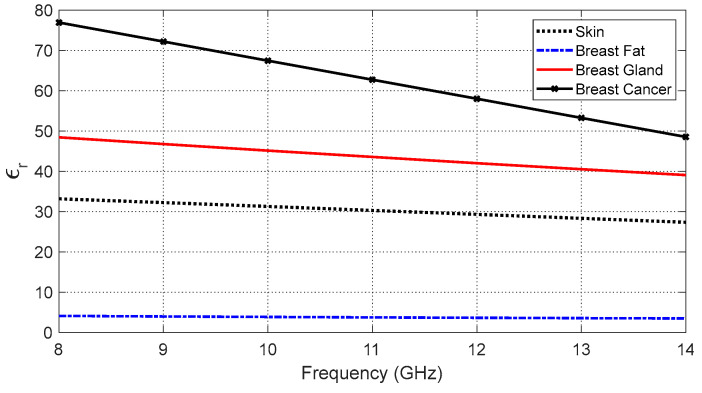
Variation in the dielectric constant of the different tissue types over the frequency band 8–14 GHz.

**Figure 11:**
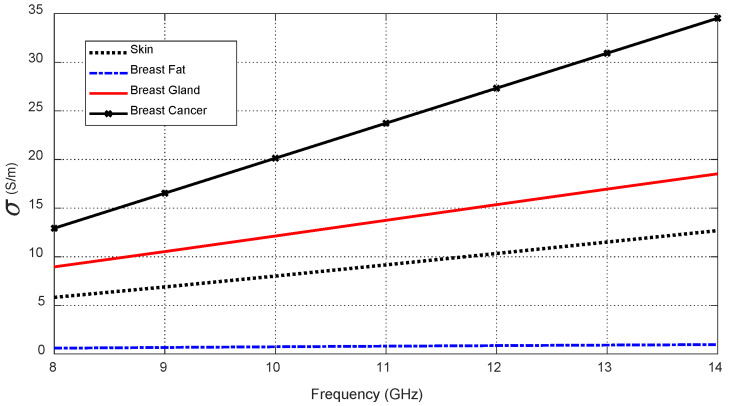
Variation in the electric conductivity of the different tissue types over the frequency band 8–14 GHz.

To simulate the antenna operation while being placed on the human breast model, the three-layer model (skin–breast fat–breast gland) can be used, as shown in Figure 8. This model is required to obtain the optimum values of the geometrical parameters of the proposed antenna design (shown in Figure 1 and Figure 2) through a complete parametric study. Also, the curved three-layer model of the human breast, shown in Figure 9, can be used to obtain the best design of the curved antenna structure. The proposed antenna is designed to operate over the frequency band 8.3–13.3 GHz. In this frequency band, the materials of the human tissues have dispersive electric properties (dielectric constant and electric conductivity) that are given by Table 3 and Table 4 and described by the curves shown in Figure 10 and Figure 11. The electric properties of the normal breast tissues are obtained from [25], whereas the properties of the cancerous breast tissues are obtained from [26]. These dispersive relations are used for the simulation of the antenna operation in the CST microwave studio^®^. The contrast between the electric properties of the normal and cancerous breast tissues can be employed to efficiently detect the cancerous tissues buried in the breast.

## 4. SAR Distribution in Human Tissues

**Figure 12:**
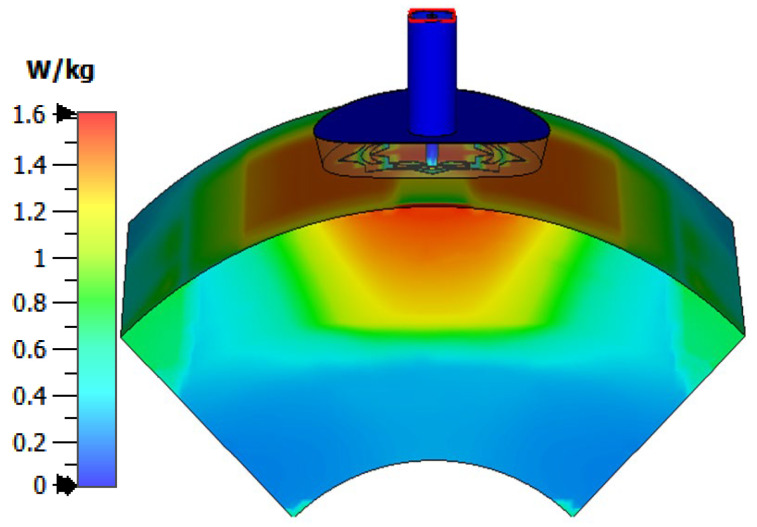
SAR distribution in the tissues of the curved breast model (radius of curvature = 5 cm) when the input power of the antenna is 8.5 dBm.

For safe exposure of electromagnetic radiation to human health, the SAR in human tissues should be limited to 1.6 W/kg [27]. The present section is concerned with presenting and discussing the numerical results for the 3D SAR distribution in the body model resulting from the curved antenna structure and the corresponding values of the antenna output power to ensure safe electromagnetic exposure. The SAR distribution in the tissues of the curved body model when the antenna is placed touching the skin is presented in Figure 12. It is shown that the maximum SAR (averaged over 1 g) does not exceed 1.6 W/kg when the antenna output power is set to 8.5 dBm (7 mW). The output power should be limited to this maximum value to ensure safe electromagnetic exposure to human health [27].

## 5. Fabrication and Experimental Results

This section is concerned with the description of antenna fabrication and the experimental measurements performed to investigate some of the antenna characteristics. Also, some of the measurements are compared with the corresponding CST^®^ simulation results for verification.

### 5.1. Antenna Fabrication

**Figure 13:**
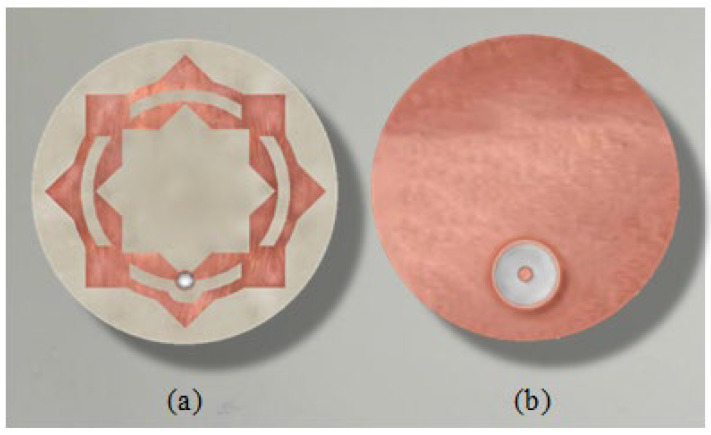
Fabricated prototype of the proposed on-skin antenna. (**a**) View of the patch side. (**b**) View of the ground side.

A prototype is fabricated for the proposed antenna (see Figure 13), with the dimensions listed in Table 1. The fabricated antenna has a flat structure and is fabricated on RO3003™ circular substrate of radius $R_{S}=5\;mm$ and height h=0.25 mm. Since this substrate is flexible, the fabricated antenna can be conformal to the human body and can be bent to take the same radius of curvature as the body surface at the region of contact.

### 5.2. Experimental Evaluation of the Reflection Coefficient

**Figure 14:**
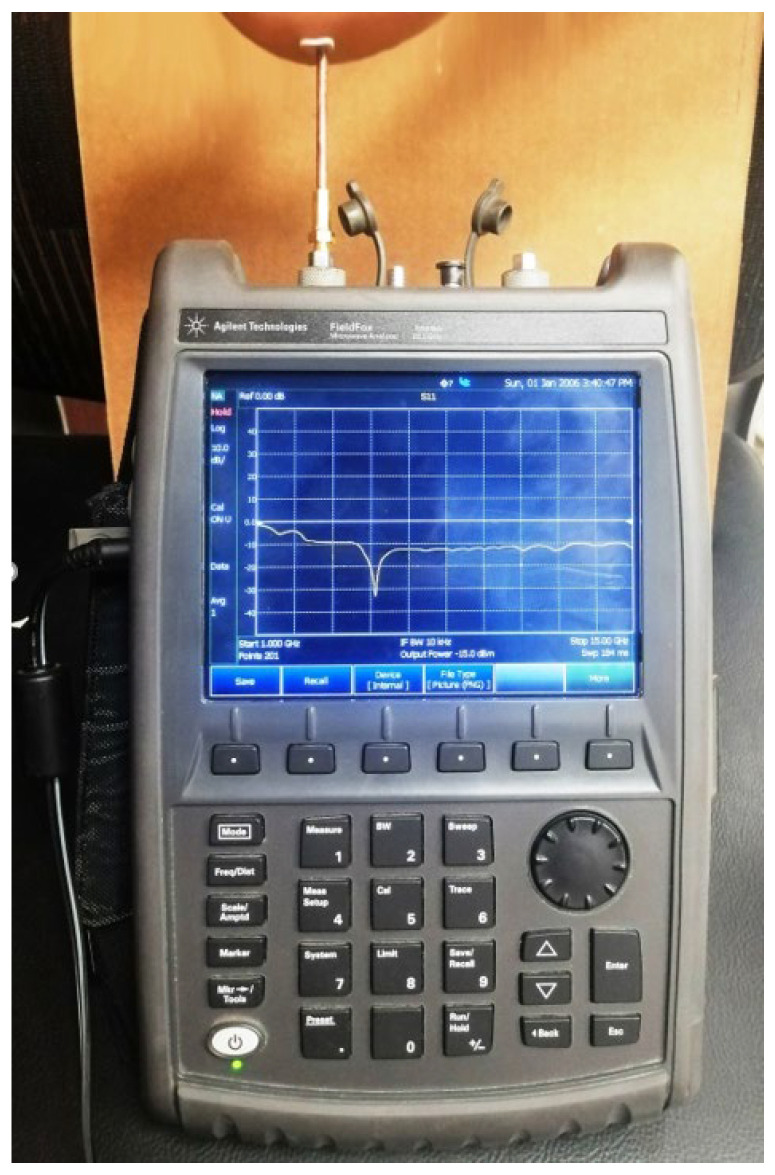
The VNA of the Agilent-FieldFox^®^ model N9918A is used to measure $S_{11}$ over the frequency band (2–26 GHz) while the antenna is being placed on the skin and bent with a radius of curvature $R_{B}\approx 5\;cm$.

The experimental setup for measuring the reflection coefficient at the antenna port is presented in Figure 14. The vector network analyzer (VNA) model Agilent-FieldFox^®^ N9918A (Keysight Technologies, Santa Clara, CA, USA) is for the measurement over the frequency range 7–15 GHz. An SMA connector is used between the antenna port and the VNA cable.

**Figure 15:**
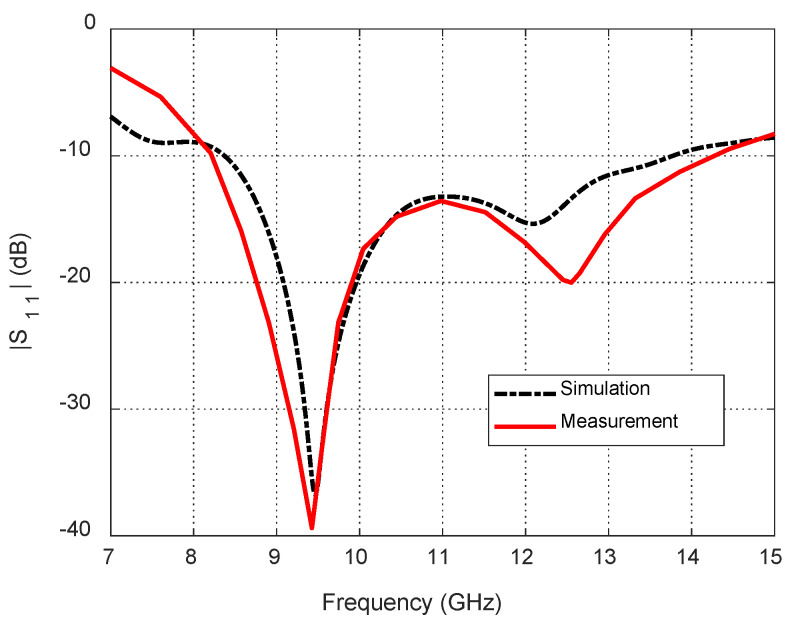
Dependence of the reflection coefficient magnitude, $\left|S_{11}\right|$, on the frequency for the blended-corner strip-dipole antenna when fed through the proposed balun.

The measured frequency response of $\left|S_{11}\right|$ is presented in Figure 15 in comparison to the results obtained by the CST^®^ simulator. The measured response shows good agreement with the results of the simulation.

## 6. Off-Body Radiation Characteristics

As mentioned before, the on-skin antenna introduced in the present work is proposed to launch electromagnetic power into human tissues and to minimize power radiated off the body (i.e., to free space). This enhances the performance of the proposed antenna for applications requiring in-body radiation, such as microwave imaging of the human tissues, cancer detection, and in-body communication with the implantable sensors in a WBAN. However, the present section is concerned with investigating off-body radiation, including the far-field parameters, such as the radiation patterns, gain, and antenna efficiency.

The radiation characteristics of the curved antenna when placed on a curved body model with a radius of curvature $R_{B}=5\;cm$, as shown in Figure 9, are numerically investigated. The antenna characteristics under investigation include the radiation pattern, antenna efficiency, directivity, and gain.

### 6.1. Radiation Patterns

**Figure 16:**
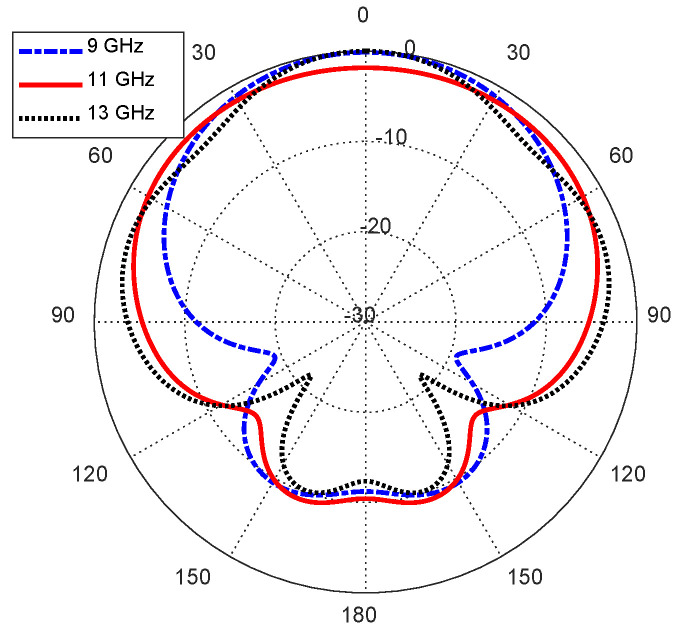
Radiation patterns of the curved antenna on the curved body model in the elevation plane $\phi =0^{{}^{\circ}}$.

When the curved antenna is placed on the skin layer of the curved human body model, as shown in Figure 9, it produces the radiation patterns presented in Figure 16 at different frequencies over the operational frequency band. Like the case of the flat antenna, the far field produced by the curved antenna is mainly directed in the free space side, which implies that the antenna output power in the direction of the human body is confined in the human tissues and does not arrive in the far zone.

### 6.2. Total and Radiation Efficiencies

**Figure 17:**
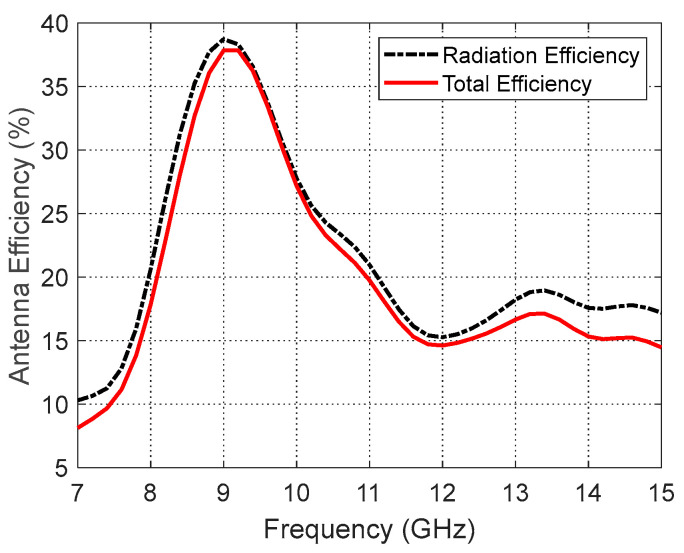
Variation in the total and radiation efficiencies of the curved antenna on a curved human model over the operational frequency band.

The frequency responses of the total and radiation efficiencies of the curved antenna when placed on a curved body model are presented in Figure 17. The radiation and total efficiencies of the curved antenna are below 20% over most of the operational frequency band. This means that most of the antenna output power (more than 80%) is confined in human tissues, which is consistent with the antenna’s operational requirement.

### 6.3. Directivity and Gain

**Figure 18:**
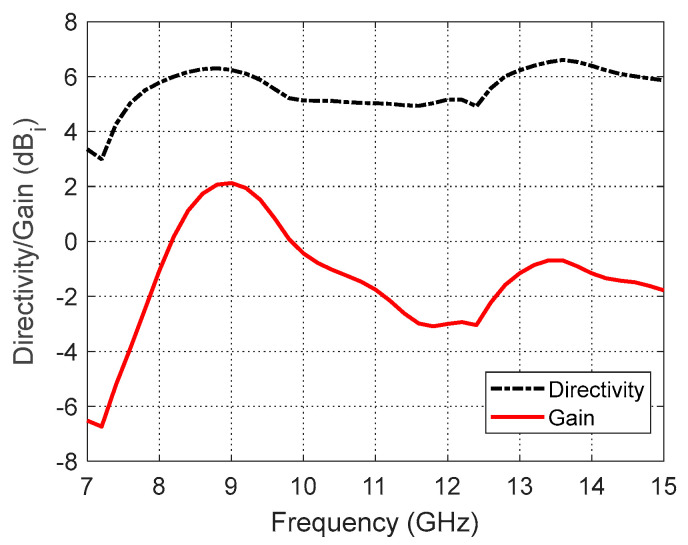
Variation in the maximum directivity and gain of the curved antenna on a curved human model over the operational frequency band.

The variations in the maximum directivity and gain of the curved antenna when placed on a curved human body with the frequency are presented in Figure 18. The gain is much lower than the directivity, which reflects the low radiation efficiency of the proposed antenna. This indicates that the curved antenna structure is capable of confining the output power in human tissues.

## 7. Comparison of the Antenna Performance with Published Work

The dimensions and characteristics of the on-body antenna proposed in the present work are compared to those of other antenna designs presented in recent publications. Table 5 provides a list of these comparisons. It is shown from the list of comparisons in Table 5 that the antenna proposed in the present work has the smallest size and the widest frequency band among those presented in the list. Moreover, it is flexible and conformal to the body without any bad effect due to bending.

## 8. Reduced Antenna System and Truncated Breast Model for Simulation of Breast Cancer Detection

### 8.1. Truncated Model of the Breast

**Figure 19:**
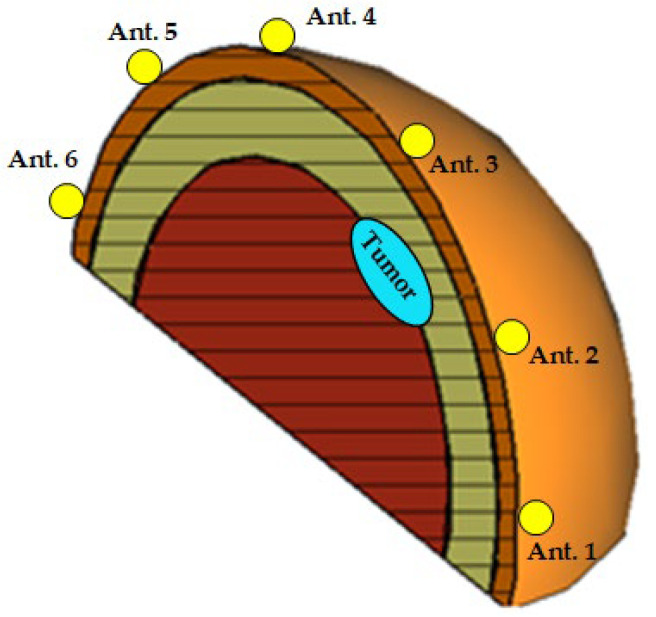
A 3D view showing vertical cross-section of the hemispherical breast model, where the radar scanning plane coincides with the tumor plane ($\varphi_{L}=\phi_{C}$). The antenna system consists of six elements uniformly distributed along the perimeter. The tumor is positioned in the mid-angular sector between antennas 2 and 3.

A three-layer hemisphere (skin–breast fat–breast glands), as shown in Figure 19 (with an embedded ellipsoidal tumor), can be used as a complete geometrical model for simulation, considering the electric properties listed in Table 3 and Table 4. Additionally, for a realistic simulation of the breast cancer detection scenario, an antenna system comprised of six antennas is placed on the surface of the hemispherical breast model, as shown in Figure 19.

The proposed antenna system is constructed as an arc array whose elements are uniformly distributed along the perimeter of the hemispherical breast model, with 30° angular separation between consecutive antennas. This arc array lies in a vertical plane defined by the azimuthal angle $\varphi_{L}$, which is adjustable via a mechanical rotator capable of a 180° rotation to scan all elevation planes (considering reciprocity).

However, extensive preliminary simulation studies revealed that, for any given tumor elevation angle $\theta_{C}$, only the subset of antennas in the immediate angular vicinity of the tumor exhibited significant perturbations in their S-parameters. For example, when $\theta_{C}=60{}^{\circ}$, primarily antennas 1–4 (with elevation angles of 15°, 45°, 75°, and 105°, respectively) show measurable changes, while antennas farther away remain largely unaffected. Furthermore, using either the group of antennas {1, 2, 3} or {2, 3, 4} provides nearly identical tumor parameter estimation accuracy compared to utilizing the full six-antenna dataset.

**Figure 20:**
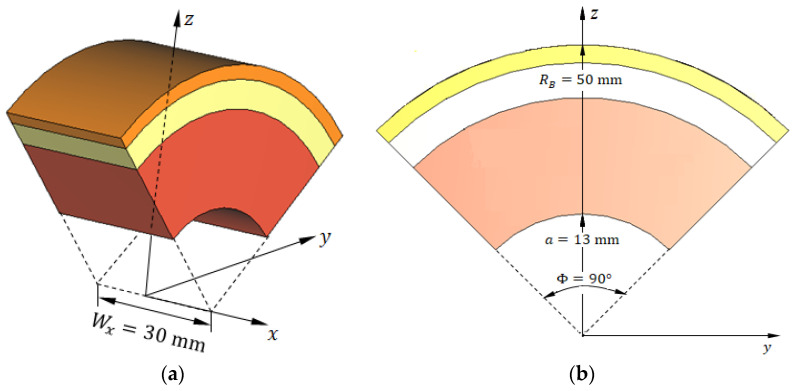
Truncated hemispherical breast model used for simulation. (**a**) Three-dimensional perspective view showing the truncated sector bounded by five surfaces. (**b**) Vertical cross-section illustrating the skin, breast fat, and glandular tissue layers, as well as the embedded ellipsoidal tumor. This reduced geometry enables focused simulation of tumor detection using three antennas while preserving the fidelity of electromagnetic interactions.

To reduce computational complexity and accelerate the simulation process without compromising detection performance, we employed a truncated breast model focusing on the most relevant angular sector and limited the antenna system to three elements. Validation experiments confirmed that this configuration produces results consistent with the full-array simulations. Specifically, the hemispherical breast model of radius $R_{B}$ is truncated to define a cylindrical sector bounded by five surfaces: (i, ii) the two parallel planes $x=-W_{x}/2$ and $x=W_{x}/2$ aligned with the principal yz plane, (iii) the cylindrical surface $z=\sqrt{a^{2}-y^{2}}$, whose axis is oriented along the x-axis, (iv, v) the two inclined planes y=−ztan(Φ/2) and y=ztan(Φ/2), perpendicular to the yz plane and intersecting along the x-axis. This truncated breast model, illustrated in Figure 20 (Figure 20a shows the 3D view; Figure 20b shows a vertical section), is configured with the following geometrical parameters: $R_{B}=50\;mm$, $W_{x}=30\;mm$, a=13 mm, and Φ=90°. This configuration enables simulation with only three antennas positioned in the immediate vicinity of the tumor instead of the complete six-antenna system. Such simplification substantially reduced computational requirements while maintaining the accuracy and generality of the training dataset used for the deep learning model.

### 8.2. Reduced Antenna System for Accurate Simulation of Breast Cancer Detection

As mentioned before, we propose to employ an arc-shaped antenna array consisting of six elements uniformly distributed along the perimeter of the hemispherical breast model. However, to reduce simulation time and efficiently demonstrate the proposed breast cancer detection methodology, a reduced portion of the complete breast model (focusing on the immediate vicinity of the tumor) is used in conjunction with only three antennas in the simulation. Additionally, the three-layer breast model (skin, breast fat, and breast gland tissues) is adopted for electromagnetic analysis.

**Figure 21:**
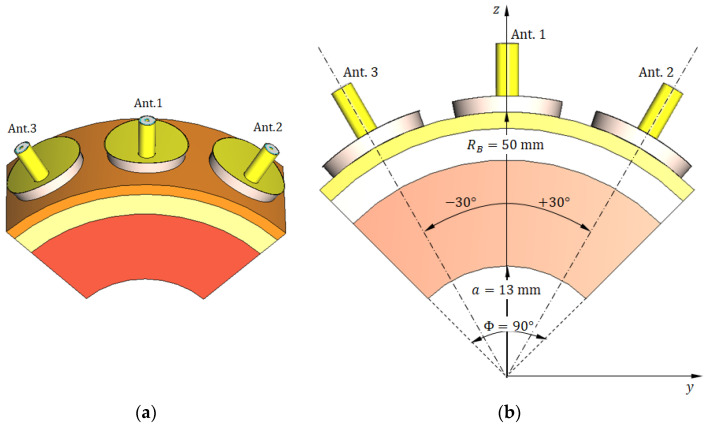
The reduced (truncated) model of the breast for simulation of breast cancer detection scenario. (**a**) A 3D view showing three antennas in direct contact with the skin surface. (**b**) Vertical section showing the three antennas with 30° angular separation and other important dimensions.

The geometric model of the truncated breast model illustrated in Figure 20 is employed to facilitate all stages of the deep learning pipeline, including feature extraction, frequency sub-band selection, construction of training datasets, validation, and performance evaluation. The antenna system used in the simulation to generate the dataset required for breast cancer detection comprises three antennas placed in direct contact with the skin surface, as shown in Figure 21. The antennas are uniformly distributed along the surface of the truncated breast model with a 30° angular separation between consecutive elements, as illustrated in Figure 21.

The rationale for this reduction in the antenna system is further detailed in Section 8.4, where antenna sensitivity analysis confirms that a three-element subset adequately captures tumor-induced perturbations.

### 8.3. Geometrical Model of the Tumor Tissues

**Figure 22:**
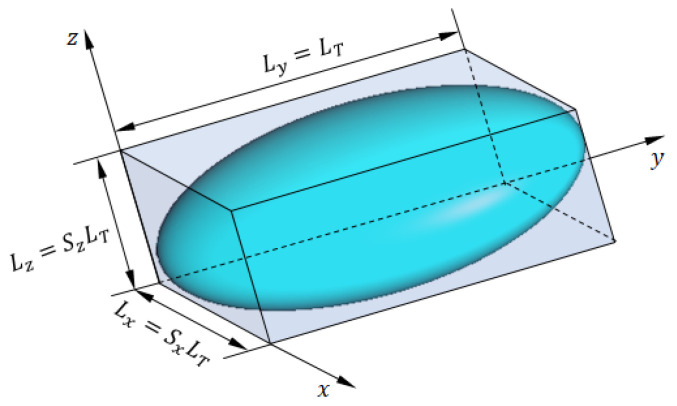
The ellipsoidal model of breast tumor tissues.

To attain as close as possible to the practical shape of the tumor tissues in the breast, it is modeled as an ellipsoid with a major length of $L_{T}$, where the major axis is directed along the y-axis, as shown in Figure 22. The bounding box of the spheroidal tumor is a cuboid whose faces are tangential to the tumor. The dimensions of this bounding box along the x-, y-, and z-axes are $L_{x}=S_{x}L_{T}$, $L_{y}=L_{T}$, and $L_{z}=S_{z}L_{T}$, respectively, where ${0<S}_{x}<1$ and ${0<S}_{z}<1$. The electric properties of the tumor tissues are listed in Table 3 and described by the curves presented in Figure 10 and Figure 11 over the frequency range 8–14 GHz. Both the geometric and electric models of the breast tumor are used in the CST simulator to obtain realistic results.

### 8.4. Antenna Sensitivity Analysis and Justification of Reduced Array

To validate the selection of a reduced three-element antenna system, we conducted extensive simulation studies to quantify the sensitivity of each antenna’s S-parameters to tumor presence as a function of tumor elevation angle $\theta_{C}$. Specifically, for each tumor position, we evaluated the mean and variance of the perturbation induced in the self and mutual S-parameters relative to the tumor-free case.

The results consistently demonstrated that only antennas within the immediate angular vicinity of the tumor exhibit significant S-parameter deviations. For example, when the tumor was located at $\theta_{C}=60{}^{\circ}$, only antennas 1–4 (with elevation angles of 15°, 45°, 75°, and 105°, respectively) showed measurable sensitivity, while antennas 5 and 6 remained largely unaffected. Similarly, for tumors positioned at $\theta_{C}=30{}^{\circ}$, antennas 1–3 were most responsive.

These findings indicate that subsets of three consecutive antennas (e.g., antennas {1, 2, 3} or {2, 3, 4}) provide nearly identical detection accuracy compared to using all six antennas. This observation supports the feasibility of using a truncated breast model with a reduced antenna system, yielding substantial reductions in simulation time and computational cost without compromising detection performance.

Let $S_{ij}^{\left(0\right)}(f)$ be the magnitude of the mutual S-parameter $S_{ij}$ without a tumor (i.e., baseline measurement) as a function of frequency (f). Let $S_{ij}^{\left(T\right)}(f)$ be the magnitude of the same S-parameter $S_{ij}$ with the tumor present at a specific position. Let $F_{ij}=f_{max}-f_{min}$ be the selected frequency sub-band for antenna pair (i,j) as determined by the proposed Attention-Based Frequency Selection (ABFS) explained later in Section 10.4.

The antenna sensitivity analysis is based on the normalized change in mutual S-parameter magnitude, $∆S_{ij}^{\left(dB\right)}$. It is calculated for each antenna pair by first computing the absolute difference between the tumor-present and baseline S-parameter magnitudes over the frequency band selected by the ABFS module. The mean difference is then normalized to the mean baseline magnitude and expressed in decibels. The detailed procedure can be described in the following steps.

Step 1: Compute the absolute difference in S-parameter magnitude over the selected frequency band as follows:

(1) $$∆S_{ij}\left(f\right)=\left|S_{ij}^{\left(T\right)}\left(f\right)-S_{ij}^{\left(0\right)}(f)\right|$$

Step 2: Compute the mean change over the selected frequency sub-band as follows:

(2) $$\mathrm{mean}(∆S_{ij})=\frac{1}{F_{ij}}\int_{f_{min}}^{f_{max}}∆S_{ij}\left(f\right)df$$

For discrete frequency samples $f_{k},\;k=1,2,\ldots ,K$, the last integral becomes

(3) $$mean(∆S_{ij})=\frac{1}{K}\sum_{k=1}^{K}\left|S_{ij}^{\left(T\right)}\left(f_{k}\right)-S_{ij}^{\left(0\right)}(f_{k})\right|$$

Step 3: Compute the mean baseline S-parameter magnitude over the same frequency band as follows:

(4) $$mean(S_{ij}^{\left(0\right)})=\frac{1}{K}\sum_{k=1}^{K}S_{ij}^{\left(0\right)}(f_{k})$$

Step 4: Compute the normalized mean change relative to the baseline mean magnitude in the same band as follows:

(5) $$∆S_{ij}^{\left(dB\right)}=20\log_{10}\left(1+\frac{mean(∆S_{ij})}{mean(S_{ij}^{\left(0\right)})}\right)$$

Table 6 presents the normalized sensitivity metric $∆S_{ij}^{\left(dB\right)}$ for all consecutive antenna pairs across representative tumor elevation angles. As observed, each tumor position predominantly affects the S-parameters measured by the antennas closest to its elevation plane. For instance, when θ=30°, ${\Delta S}_{21}$ exhibits the highest response (0.88 dB), whereas the distal antenna pairs remain largely insensitive (<0.1 dB). This confirms that only a subset of antennas is required to achieve robust detection accuracy, supporting the truncation strategy described in Section 8.

**Figure 23:**
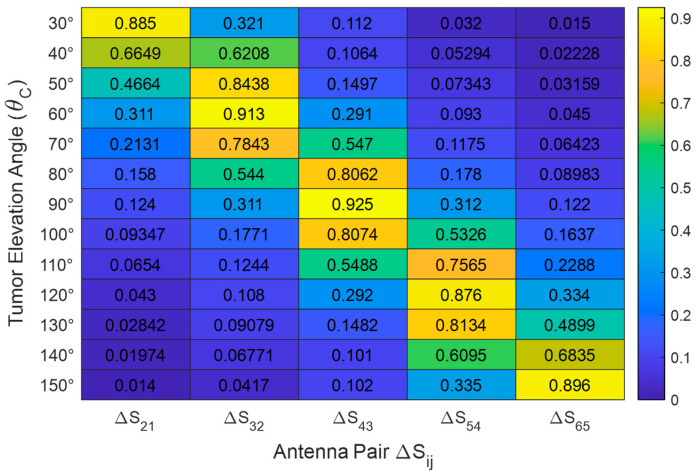
Sensitivity profiles showing the normalized change in S-parameter magnitude (${∆S}_{ij}$) across all six antennas for selected tumor positions.

Figure 23 illustrates representative sensitivity profiles, showing the normalized change in S-parameter magnitude, $∆S_{ij}^{\left(dB\right)}$, across all six antennas for selected tumor positions. This change is calculated by averaging the changes in each mutual S-parameter over the frequency range that is selected by applying the proposed ABFS. For example, when $\theta_{C}=70{}^{\circ}$, the optimal antenna triplet is {2, 3, 4}, as ${∆S}_{32}$ and ${∆S}_{43}$ exhibit the highest magnitudes compared to the other antenna pairs. This subset can be employed for dataset generation and feature extraction without any appreciable loss in detection accuracy. For other elevation angles, such as $\theta_{C}=90{}^{\circ}$, either the triplet {2, 3, 4} or {3, 4, 5} can similarly be used in place of the complete six-antenna system. This selective approach significantly reduces computational cost while preserving the fidelity of the generated training data and the performance of the proposed detection model.

For clarity and convenience, the selected three antennas will hereafter be designated as follows: the central antenna (nearest one to the tumor) is referred to as Ant. 1, the right-hand antenna as Ant. 2, and the left-hand antenna as Ant. 3 (see Figure 21). All simulation results presented in the remainder of this paper will use these labels to identify the antennas. Hereafter, the coordinate system employed to allocate the tumor within the truncated breast model will be that shown in Figure 21b.

### 8.5. Characteristics of the Antenna System on the Breast Model

**Figure 24:**
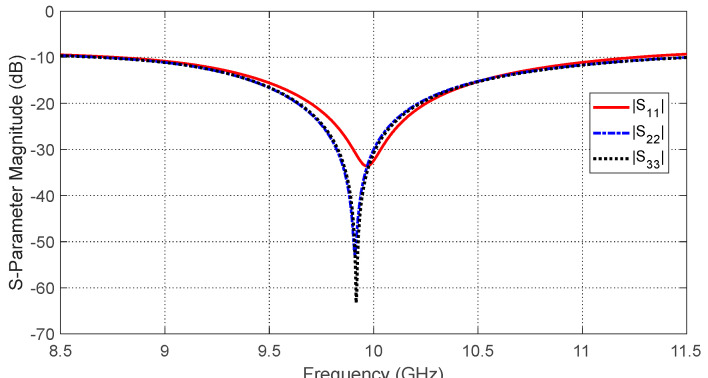
Frequency response of the magnitudes of the self-scattering parameters $\left|S_{11}\right|$, $\left|S_{22}\right|$, and $\left|S_{33}\right|$, for the antenna system placed on the breast model (free from tumor), as shown in Figure 21.

**Figure 25:**
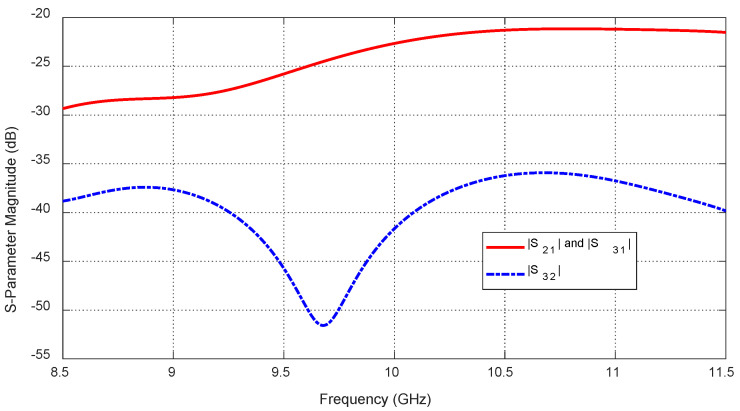
Frequency response of the magnitudes of the mutual-scattering parameters $\left|S_{21}\right|$, $\left|S_{31}\right|$, and $\left|S_{32}\right|$, for the antenna system placed on the breast model (free from tumor), as shown in Figure 21.

The frequency responses of the magnitudes of the self-scattering parameters of the three antennas, $\left|S_{11}\right|$, $\left|S_{22}\right|$, and $\left|S_{33}\right|$, are shown in Figure 24 over the frequency range 8.5–11.5 GHz that is selected for simulation of the breast cancer detection. The simulation results show that the impedances of the three antennas are matched over the entire frequency band of the simulation with sharp resonances near the central frequency (10 GHz). Figure 25 shows the frequency responses of the magnitudes of the three mutual S-parameters $\left|S_{21}\right|$, $\left|S_{31}\right|$, and $\left|S_{32}\right|$. The results show that, in the absence of the tumor, the mutual coupling coefficients between the consecutive antennas ($\left|S_{21}\right|$ and $\left|S_{31}\right|$) are less than −20 dB over the entire frequency band of operation, whereas the mutual coupling coefficient $\left|S_{32}\right|$ is less than −35 dB.

## 9. Simulation Results for Training Dataset and Feature Extraction

**Figure 26:**
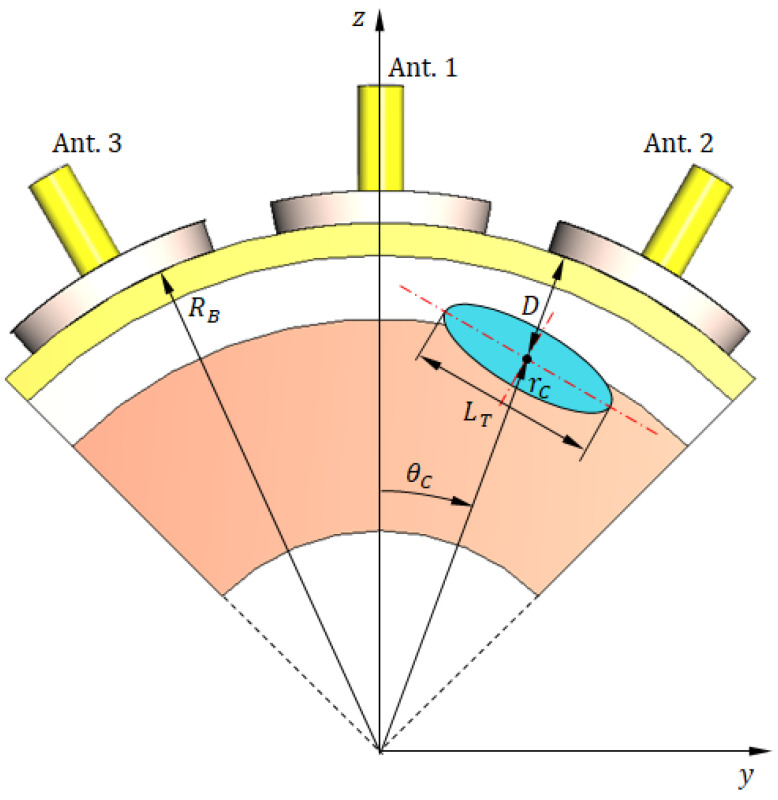
The aim of the tumor target detection algorithm is to accurately identify the embedded tumor location ($r_{C},\phi_{C}$) and size ($L_{T}$) using the mutual coupling parameters $\left|S_{21}\right|$ and $\left|S_{31}\right|$, obtained from the antenna system over the frequency band 8.5–11.5 GHz.

The process of breast cancer detection proposed in the present work can be described in view of Figure 26, where the tumor to be detected is embedded at the location ($r=r_{C},{\theta =\theta }_{C},\phi =\varphi_{R}$), where ϕ= $\varphi_{R}$ is the azimuth plane of the microwave radar (antenna system). The aim of the proposed tumor detection algorithm is to accurately estimate the tumor size ($L_{T})$ and the tumor location ($r_{C},\theta_{C}$) from the measured magnitudes of the self S-parameters ($\left|S_{11}\right|$, $\left|S_{22}\right|$, and $\left|S_{33}\right|$) and the mutual S-parameters ($\left|S_{21}\right|$, $\left|S_{31}\right|$, and $\left|S_{32}\right|$). The depth (D) of the center point of the tumor ellipsoid below the skin surface can be used as an alternative to $r_{C}$ for determining the tumor location within the breast model ($D=R_{B}-r_{C}$).

The proposed algorithm relies on understanding the dependence of the six S-parameters mentioned above (both self and mutual) on each of the parameters $L_{T}$, D, and $\theta_{C}$. This can be achieved by performing a complete parametric study, where the three parameters to be estimated are varied within their geometric limiting values determined by the region of detection, as shown in Figure 26. Thus, the following constraint is applied while studying the effect of varying the tumor angular position $\theta_{C}$.

This figure illustrates the simulation setup, where the tumor is placed at ($r=r_{C},{\theta =\theta }_{C},\phi =\varphi_{R}$), with $\varphi_{R}$ denoting the azimuthal plane of the antenna array. The objective is to estimate tumor size $L_{T}$ and location ($r_{C},\theta_{C}$) from the measured magnitudes of the self S-parameters $\left|S_{11}\right|$, $\left|S_{22}\right|$, and $\left|S_{33}\right|$ and mutual S-parameters $\left|S_{21}\right|$, $\left|S_{31}\right|$, and $\left|S_{32}\right|$. The depth $D=R_{B}-r_{C}$ provides an equivalent measure of tumor position.

The tumor parameters are subjected to the following constraints.

(6) $$-30{}^{\circ}\leq \theta_{C}\leq 30{}^{\circ}$$

(7) $$R_{B}-L_{z}/2\leq D\leq L_{z}/2$$

(8) $$0<L_{T}<20\;mm$$

### 9.1. Dependence of the S-Parameters on the Angular Position of the Tumor

**Figure 27:**
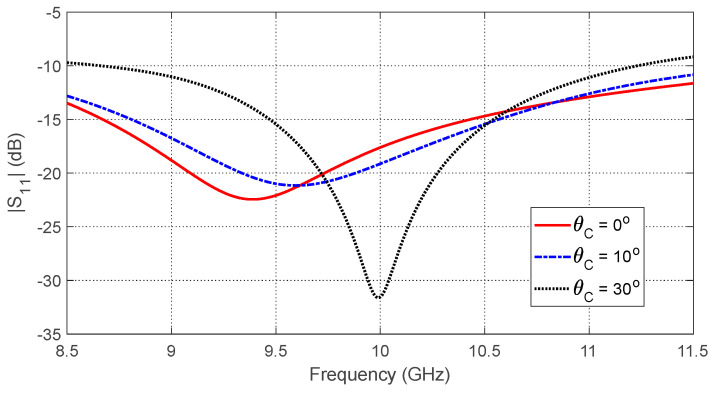
Variation in the frequency response of $\left|S_{11}\right|$, for different values of the angular position, $\theta_{C}$, of an ellipsoidal tumor of major length $L_{T}=12\;mm$ embedded at a depth of D=20 mm below the skin surface of a hemispherical breast model.

**Figure 28:**
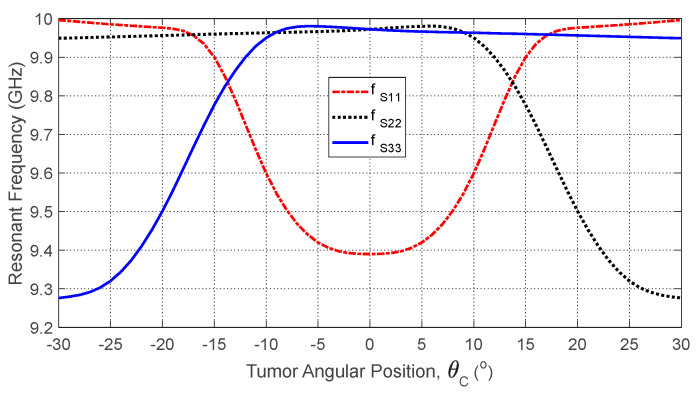
Variation in the resonant frequencies corresponding to the minima of the self S-parameters $\left|S_{11}\right|$, $\left|S_{22}\right|$*,* and $\left|S_{33}\right|$ with varying the angular position, $\theta_{C}$, of an ellipsoidal tumor of major length $L_{T}=12\;mm$ embedded at a depth of D=20 mm below the skin surface of a hemispherical breast model.

**Figure 29:**
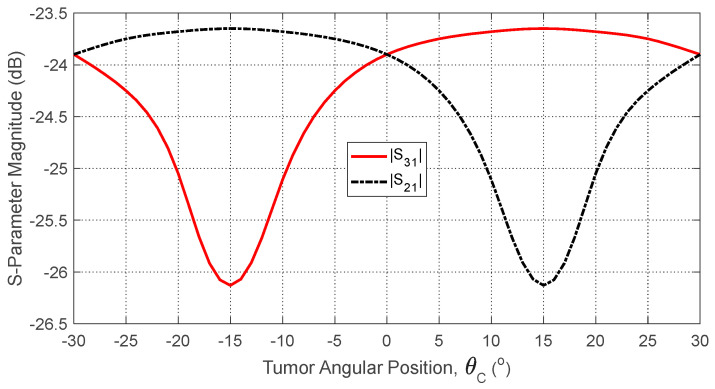
Dependence of the mutual S-parameters $\left|S_{21}\right|$ and $\left|S_{31}\right|$ (averaged over the frequency range 10–11 GHz) on the angular position of a spheroidal tumor of length $L_{T}=14\;mm$ embedded at a depth of D=20 mm below the skin surface.

The tumor’s angular position $\theta_{C}$ affects its proximity to the antennas, shifting their resonant frequencies due to local changes in dielectric properties. Figure 27 shows how the self S-parameter $\left|S_{11}\right|$ varies with $\theta_{C}$. As the tumor moves away from Antenna 1, the resonant frequency increases. Figure 28 plots the resonant frequencies of $\left|S_{11}\right|$, $\left|S_{22}\right|$, and $\left|S_{33}\right|$ versus $\theta_{C}$, demonstrating their utility as features for estimating angular position. Similarly, Figure 29 shows how mutual S-parameters $\left|S_{21}\right|$, $\left|S_{31}\right|$, and $\left|S_{32}\right|$ vary with $\theta_{C}$, providing complementary information.

### 9.2. Dependence of the S-Parameters on the Tumor Depth Below the Skin

**Figure 30:**
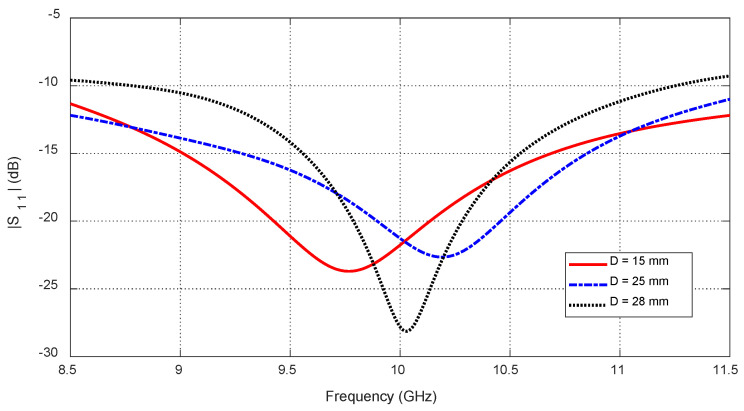
Frequency responses of $\left|S_{11}\right|$ for different values of the depth, D, beneath the skin surface of a hemispherical breast model at which an ellipsoidal tumor of major length $L_{T}=12\;mm$ is embedded at the angular position $\theta_{C}=0{}^{\circ}$.

**Figure 31:**
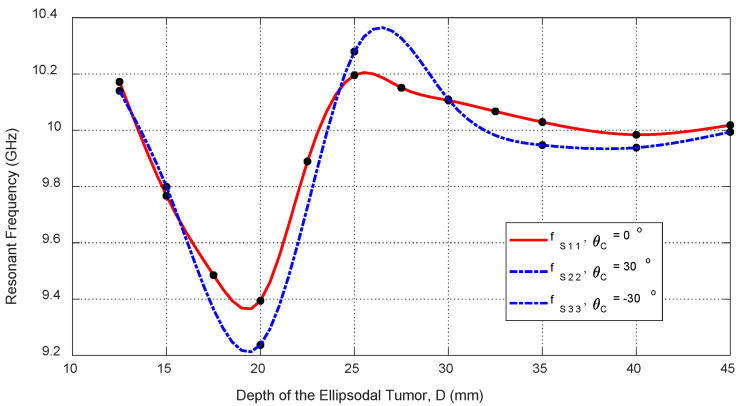
Variation in the resonant frequencies corresponding to the minima of the self S-parameters $\left|S_{11}\right|$, $\left|S_{22}\right|$, and $\left|S_{33}\right|$ with varying the depth, D, beneath the skin surface of a hemispherical breast model at which an ellipsoidal tumor of major length $L_{T}=12\;mm$ is embedded at the angular positions $\theta_{C}=0{}^{\circ}$, 30°, and −30°, respectively.

**Figure 32:**
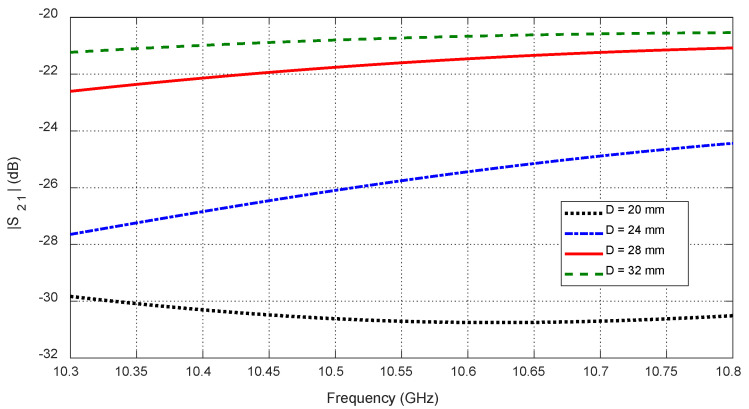
Variation in the mutual scattering parameter $\left|S_{21}\right|$ with the frequency for different values of the depth, D, at which a spheroidal tumor of length $L_{T}=8\;mm$ is embedded below the skin surface at the angular position $\theta_{C}=15{}^{\circ}$.

**Figure 33:**
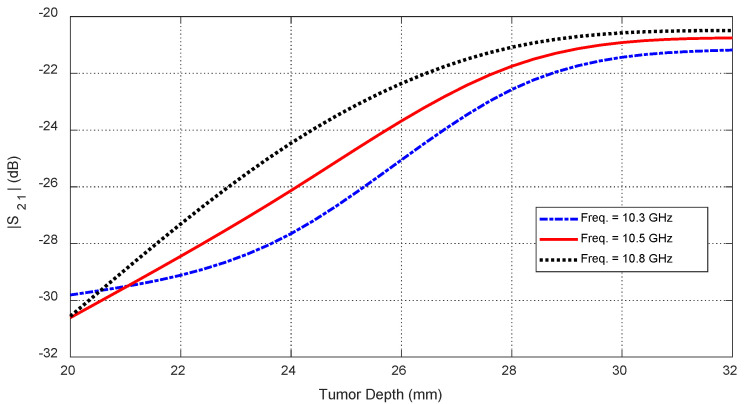
Dependence of the mutual scattering parameter $\left|S_{21}\right|$ on the depth D below the skin surface at which an ellipsoidal tumor of major length $L_{T}=8\;mm$ is embedded at the angular position $\theta_{C}=15{}^{\circ}$.

**Figure 34:**
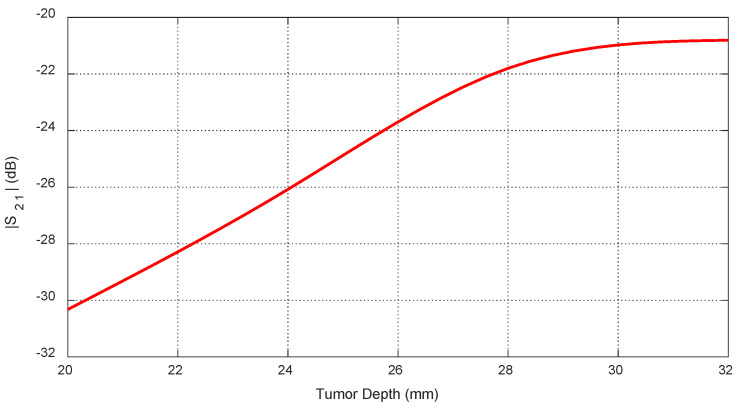
Dependence of $\left|S_{21}\right|$, averaged over the frequency range 10–11 GHz, on the depth, D, beneath the skin surface, where a spheroidal tumor of length $L_{T}=14\;mm$ is embedded at an angular position $\theta_{C}=15{}^{\circ}$.

Tumor depth D affects both self and mutual S-parameters, especially when the tumor is aligned beneath or between antennas. Figure 30 shows the frequency response of $\left|S_{11}\right|$ for different depths. Figure 31 plots the corresponding resonant frequencies versus D, indicating that shallower tumors induce more pronounced shifts. Figure 32, Figure 33 and Figure 34 illustrate how $\left|S_{21}\right|$ varies with depth, confirming that averaged mutual S-parameters are also effective for estimating tumor depth.

### 9.3. Dependence of the S-Parameters on the Tumor Size

**Figure 35:**
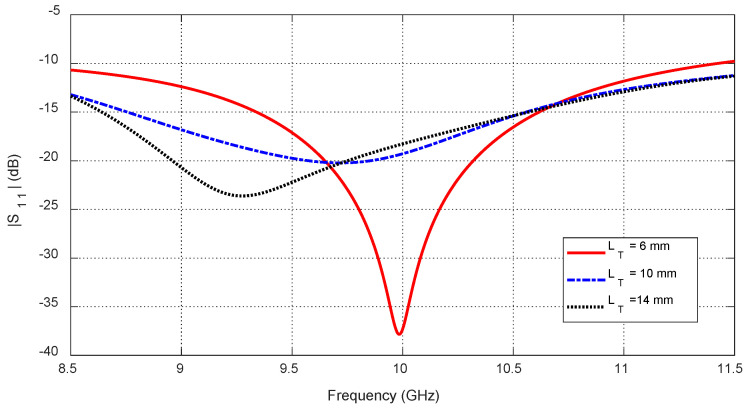
The frequency responses of $\left|S_{11}\right|$ for different values of the major length ($L_{T}$) of an ellipsoidal tumor embedded at a depth of D=20 mm beneath the skin surface of a hemispherical breast model at the angular position $\theta_{C}=0$.

**Figure 36:**
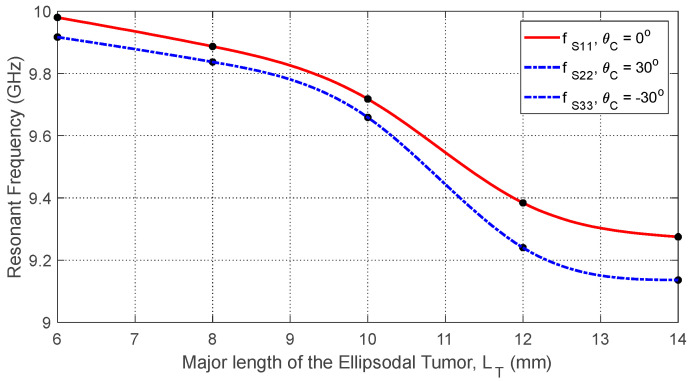
Variation in the resonant frequencies corresponding to the minima of the self S-parameters $\left|S_{11}\right|$, $\left|S_{22}\right|$, and $\left|S_{33}\right|$ with varying the major length ($L_{T}$) of an ellipsoidal tumor embedded at a depth of D=20 mm beneath the skin surface of a hemispherical breast model at the angular positions $\theta_{C}=0{}^{\circ}$, 30°, and −30°, respectively.

**Figure 37:**
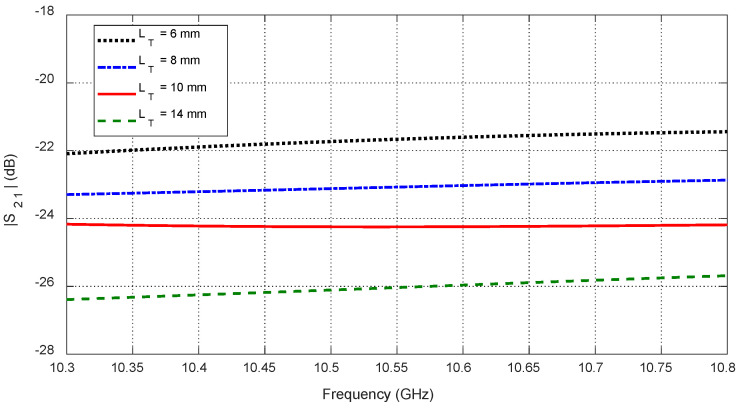
Variation in the mutual scattering parameter $\left|S_{21}\right|$ with the frequency for different values of a spheroidal tumor length, $L_{T}$, when embedded at the angular position $\theta_{C}=15{}^{\circ}$ and depth D=20 mm below the skin surface.

**Figure 38:**
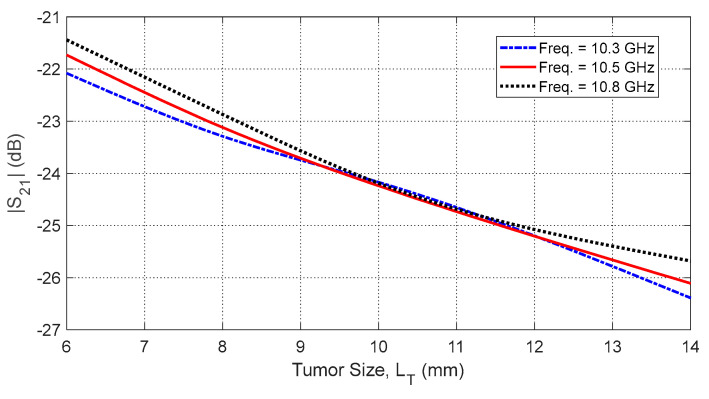
Dependence of the mutual S-parameter $\left|S_{21}\right|$ on the major length, $L_{T}$, of an ellipsoidal tumor embedded at the angular position $\theta_{C}=15{}^{\circ}$ and depth D=20 mm beneath the skin surface.

**Figure 39:**
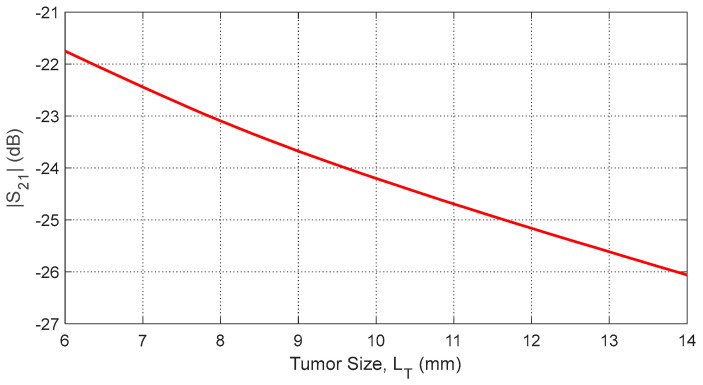
Dependence of $\left|S_{21}\right|$, averaged over the frequency range 10–11 GHz, on the length, $L_{T}$, of a spheroidal tumor embedded at angular position $\theta_{C}=15{}^{\circ}$ and depth D=20 mm beneath the skin surface.

Increasing tumor size $L_{T}$ further alters the S-parameters due to the larger dielectric perturbation. Figure 35 and Figure 36 show how the resonant frequencies of self S-parameters decrease as $L_{T}$ increases. Figure 37, Figure 38 and Figure 39 present the dependence of mutual S-parameters $\left|S_{21}\right|$ on the tumor size, demonstrating that these features can reliably indicate tumor dimensions.

## 10. Outlines of the Proposed Deep Learning Model

The proposed deep learning framework leverages microwave radar-based S-parameter data to estimate critical parameters of the ellipsoidal tumor embedded within the heterogeneous hemispherical breast model: its angular position ($\theta_{C}$) with the vertical axis of the hemispherical breast, its depth (D) beneath the skin surface, and its major length ($L_{T}$). Given the observed frequency-dependent impact of these parameters on self and mutual S-parameters, a multi-branch neural network is designed to process distinct feature sets derived from selected frequency bands. A key innovation in this model is the integration of an ABFS module, which dynamically identifies optimal frequency sub-bands for feature extraction, thereby enhancing the model’s ability to generalize across varying tumor conditions [1,2].

Unlike differential imaging techniques that require a healthy baseline for subtraction, our approach uses absolute S-parameter magnitudes as input. The deep neural network is trained to learn direct mappings between mutual coupling patterns and tumor parameters (azimuth, elevation, distance, and size), without reference to healthy tissue data. This design ensures practical applicability, as healthy S-parameters are not available in real-world screening scenarios. The use of a simplified three-antenna subset was based on a sensitivity analysis, identifying antenna pairs that contribute most to localization accuracy while minimizing system complexity.

### 10.1. Overview of the Proposed Deep Learning Model

The proposed deep learning model for breast cancer detection can be divided into two phases: learning (training) and detection (prediction). During learning, the model discovers and learns the most useful features fed at the input layer of the neural network. During detection (after learning), the model uses the learned features to make predictions about new tumor cases and provides the estimated tumor parameters at the output layer of the neural network.

During learning (training phase),

− The deep learning model automatically extracts features from the input data (curves of self and mutual S-parameters versus frequency) while being trained on labeled tumor parameters ($\theta_{C}$, D, $L_{T}$);
− The model identifies patterns, such as resonant frequency shifts and magnitude variations, that correlate with tumor parameters;
− This process is implicit; the model refines the features layer by layer through back propagation and optimization.

During tumor detection (after learning, inference phase),

− The model applies the learned features to new, unseen S-parameter data to estimate tumor parameters ($\theta_{C}$, D, $L_{T}$);
− At this stage, feature extraction is not an active learning process; the model simply processes the new input using the features it has already learned during training.

### 10.2. Dataset Construction

The dataset required for the deep learning model proposed for breast cancer detection consists of simulated S-parameters of the antenna system shown in Figure 26 over the frequency range 8.5–11.5 GHz.

#### 10.2.1. Simulation of S-Parameters for Dataset Generation

The magnitudes of the self S-parameters ($\left|S_{11}\right|$, $\left|S_{22}\right|$, $\left|S_{33}\right|$) represent the fraction of the microwave power reflected back to the feeding ports of the antennas. The magnitudes of the mutual S-parameters ($\left|S_{21}\right|$, $\left|S_{31}\right|$, $\left|S_{32}\right|$) represent the fractions of the power transmitted between the antennas of each pair. These parameters are obtained from the CST simulator employing the antenna system comprised of three antennas mounted to the truncated model of the hemispherical breast phantom with an embedded ellipsoidal tumor, as shown in Figure 26. Examples of the frequency dependencies of the self S-parameters and their variation with the tumor parameters are presented in Figure 27, Figure 30 and Figure 35. The dependencies of the resonant frequency for each of these self S-parameters on the tumor parameters are presented in Figure 28, Figure 31 and Figure 36. Examples of the frequency dependencies of the mutual S-parameters and their variation with the tumor parameters are presented in Figure 28, Figure 32, Figure 33, Figure 37 and Figure 38. The dependencies of the frequency-averaged magnitude of these mutual S-parameters on the tumor parameters are presented in Figure 29, Figure 34 and Figure 39. The parameters of the embedded ellipsoidal tumor (major length $L_{T}$, angular position $\theta_{C}$, and depth D beneath the skin surface) are varied systematically across multiple, generating a dataset covering a broad frequency range (8.5–11.5 GHz). Such variations are constrained by the inequalities given by (6), (7), and (8).

#### 10.2.2. Construction of Feature Vectors for Each Tumor Case

The data structure of the extracted features comprises 15 vectors that consist of the resonant frequencies extracted from the self S-parameters $|S_{11}|$, $|S_{11}|$, and $|S_{33}|$ as functions of the tumor angular position $\theta_{C}$, depth D, and size $L_{T}$, along with the frequency-averaged mutual S-parameters $|\bar{S_{21}}|$ and $|\bar{S_{31}}|$, as functions of the same parameters. Thus, the dataset comprises 125 tumor cases generated by systematically varying the tumor parameters across 5 discrete values of $\theta_{C}$, 5 depths D, and 5 sizes $L_{T}$, resulting in a 5×5×5 combination grid. For each case, the 15 feature vectors were extracted to serve as model inputs. Specifically, for each of the feature vectors, 25 curves were generated to cover all combinations of the two tumor parameters while sweeping the third parameter. For example, consider covering all combinations of five angular positions $\theta_{C}$ and five tumor sizes $L_{T}$, while sweeping the depth D across its five discrete values. Each curve was fitted to a smooth function, and 20 evenly spaced samples were obtained from the fitted curve, yielding 500 total values per feature (25 curves × 20 samples). Consequently, each tumor case is represented as a matrix of shape 15 features × 500 points.

### 10.3. Feature Engineering

Feature engineering is the process of using domain knowledge to select, modify, or create new input features from raw data that can better represent the underlying patterns for a machine learning model. The goal is to transform the raw data into a set of features that can help the model learn more effectively and make better predictions. By performing feature engineering, the deep learning model is essentially guided to focus on the most informative aspects of the data, which will lead to a more efficient model and more accurate predictions.

In the proposed model for breast cancer detection, feature engineering involves the process of identifying and preparing key data points (features) from the S-parameters (both self and mutual) that can be used to predict tumor parameters (size, position, and depth). These features can be related to different characteristics of the tumor and its interaction with the electromagnetic waves from the antenna system.

#### 10.3.1. Feature Selection

The most relevant features from the S-parameters that can help predict the tumor characteristics are identified. The dependence of the resonant frequency corresponding to the minimum of $\left|S_{11}\right|$ (for example) on the tumor parameters (size and location) are presented in Figure 29, Figure 31 and Figure 36. These are useful to extract key features from the data related to the frequency response of the self-scattering parameters. The feature selection involves selecting the frequency bands that show the highest correlation with each tumor parameter (size, angular position, and depth). Certain frequency ranges in the self S-parameters are more indicative of tumor parameters; for instance, the frequency range (9–10 GHz) is suitable to extract critical features from the frequency responses of the self S-parameters ($\left|S_{11}\right|$, $\left|S_{22}\right|$, and $\left|S_{33}\right|$) related to the angular position ($\theta_{C}$) of the tumor, depth (D) beneath the skin surface, and major length of the tumor ellipsoid ($L_{T}$), as illustrated in Figure 27, Figure 30 and Figure 35, respectively. Other frequency ranges are better for indicating the tumor parameters using mutual S-parameters. For instance, the frequency range (10–11 GHz) is suitable to extract key features from the frequency-averaged mutual S-parameters between consecutive antennas ($\left|S_{21}\right|$ and $\left|S_{31}\right|$) related to the tumor parameters $\theta_{C}$, D, and $L_{T}$, as illustrated in Figure 29, Figure 34 and Figure 39, respectively.

#### 10.3.2. Data Segmentation

The data from which the features are extracted can be segmented according to the type of S-parameters over the corresponding frequency bands (i.e., self and mutual S-parameters). Also, the data can be segmented according to the spatial distribution of the antennas for which the S-parameters are measured (i.e., segmented as $\left|S_{11}\right|$, $\left|S_{22}\right|$, and $\left|S_{33}\right|$ data). Additionally, the data can be segmented according to the range of the tumor parameter to be estimated. For instance, to extract key features related to the tumor angular position ($\theta_{C}$) when it lies within the range $-45{}^{\circ}<\theta_{C}<-15{}^{\circ}$, the shift of the resonant frequency extracted from the frequency behavior of $\left|S_{33}\right|$ over the frequency band (9–10 GHz) can be used. Similarly, when $\theta_{C}$ lies within the ranges $-15{}^{\circ}<\theta_{C}<15{}^{\circ}$, and $15{}^{\circ}<\theta_{C}<45{}^{\circ}$, key features related to this parameter can be extracted from the frequency behavior of $\left|S_{11}\right|$ and $\left|S_{22}\right|$, respectively, over the same frequency band.

#### 10.3.3. Feature Correlation

The features for estimation of multiple tumor parameters may be correlated; for instance, the frequency behavior of $\left|S_{31}\right|$ is used to extract features for estimating the tumor depth (D) when the tumor angular position ($\theta_{C}$) lies in the range $-30{}^{\circ}<\theta_{C}<0{}^{\circ}$, and the frequency behavior of $\left|S_{21}\right|$ is used to extract features for estimating D when $\theta_{C}$ lies in the range $0{}^{\circ}<\theta_{C}<30{}^{\circ}$ (see Figure 32, Figure 33 and Figure 34). Thus, the features extracted from the mutual S-parameter data for estimating the tumor parameters D and $\theta_{C}$ are correlated.

#### 10.3.4. Feature Transformation

Sometimes the data representing the various S-parameters need to be transformed to make them more useful for deep learning. This can involve normalization, scaling, or applying mathematical transformations. In the proposed deep learning model for breast cancer detection, it is useful to normalize the self S-parameters ($\left|S_{11}\right|$, $\left|S_{22}\right|$, and $\left|S_{33}\right|$) across different frequency bands to the maximum value of the largest self S-parameter magnitude so that the model treats them in a consistent scale. Also, it is useful to normalize the mutual S-parameters ($\left|S_{21}\right|$, $\left|S_{31}\right|$, and $\left|S_{32}\right|$) to their corresponding values when the tumor is not present within the breast model (tumor-free S-parameters of the antenna system). Another data transform applied to obtain useful features in the workflow of the proposed deep learning model is applied to extract the resonant frequency from the frequency behavior of the self S-parameters. This transform is applied to the frequency dependence curves of $\left|S_{11}\right|$, $\left|S_{22}\right|$, and $\left|S_{33}\right|$ to obtain the feature curves representing the relations between resonant frequencies and the tumor parameters $\theta_{C}$, D, and $L_{T}$, as shown in Figure 28, Figure 31 and Figure 36, respectively. Another transform is that applied to perform averaging of the mutual S-parameters $\left|S_{21}\right|$ and $\left|S_{31}\right|$ over the frequency range (10–11 GHz) to produce the feature curves describing the dependence of the frequency-averaged mutual S-parameters on the tumor parameters $\theta_{C}$, D, and $L_{T}$, as shown in Figure 29, Figure 34 and Figure 39, respectively.

#### 10.3.5. Feature Creation

In the workflow of the proposed deep learning model, new features are created by combining existing features. For instance, higher-order statistics like the mean, variance, and gradient are extracted from certain S-parameters to provide useful features that indicate the region of correlation within which a specific feature curve is useful for the estimation of some tumor parameter. For example, the relation between the tumor depth (D) and the resonant frequencies corresponding to the different self S-parameters plotted in Figure 31 shows high correlation, and hence, a large variance within the range of the tumor depth is defined as 10 mm<D<30 mm, which is the range of applicability of this feature.

#### 10.3.6. Handling Missing Data

In the proposed deep learning model, the concepts of antenna reciprocity and geometrical symmetry of a breast model are exploited to complete some missing data to complete the S-parameters. Feature engineering includes handling missing data by either imputing values or excluding features that are incomplete. For instance, if some S-parameters are required within certain frequency bands and not available in the simulation results, the missing values are imputed using the mean or median of the other data points.

### 10.4. Attention Mechanisms

In a traditional neural network, every input feature is treated with equal importance. However, in many real-world tasks, some features are more informative or relevant to the task than others. The attention mechanism aims to assign different “weights” or “scores” to different features in the input, allowing the model to focus more on the important ones and less on the irrelevant ones.

In self-attention, each feature (e.g., frequency band or antenna parameter) is compared with all others, and the model decides which ones are more important to focus on. This helps the model capture long-range dependencies or correlations between input features.

In the proposed model for breast cancer detection, we are working with self and mutual S-parameters over a frequency range. The goal is to extract meaningful features from these parameters to estimate tumor characteristics (angular position, depth, and size). Since different frequency bands may capture different tumor characteristics, an attention mechanism can be used to select and emphasize the most relevant frequency bands for each tumor parameter, based on the task at hand.

#### 10.4.1. Overview of Attention in Feature Extraction

*Frequency-Specific Feature Extraction:*

− Self S-parameters (|$S_{11}$|, |$S_{11}$|, and |$S_{33}$|): Capture variations due to tumor-induced impedance mismatches, particularly affecting the resonant frequency shift of each antenna [6].
− Mutual S-parameters (|$S_{21}$| and |$S_{31}$|): Reflect tumor-induced perturbations in signal transmission between antennas, influenced by tumor size, depth beneath the skin surface and angular position [7].

Given that different tumor parameters predominantly affect distinct frequency bands, a naive feature selection approach (using the entire frequency spectrum) may introduce redundancy and degrade prediction accuracy. To address this, we employ an attention-based frequency selection model to optimize the feature space [6].

*Attention-Based Frequency Selection (ABFS) Model:*

The ABFS module enhances feature selection by assigning attention weights to different frequency sub-bands, prioritizing those that contribute most to tumor parameter estimation [17]. The mechanism operates as follows:

− Frequency Band Partitioning: The full spectrum is divided into discrete sub-bands (e.g., 8–9 GHz, 9–10 GHz, and 10–11 GHz) [18].
− Self-Attention Mechanism: A trainable attention layer assigns adaptive weights to each sub-band, reinforcing contributions from the most relevant frequency components [19].
− Feature Embedding and Selection: Frequency-weighted features are concatenated into high-dimensional embeddings, ensuring optimal feature representation for tumor parameter estimation [2,7]. The self and mutual S-parameters are mapped to a high-dimensional space where patterns related to tumor parameters (depth, position, and size) are better captured. The deep learning model learns these embeddings to improve tumor detection accuracy.

Mathematically, given an input S-parameter feature vector X, the ABFS model computes the following:

(9) $$\tilde{X}=\sum_{n=1}^{N}\alpha_{n}X_{n}$$

where $\alpha_{n}$ represents the learned attention weight for the $n^{th}$ frequency sub-band, and $\tilde{X}$ is the optimized feature representation [1,6].

#### 10.4.2. Comparison with Classical Attention Mechanisms

The proposed ABFS introduces *branch-specific attention and feature separation*, which are absent in conventional global attention mechanisms. Each branch of the network independently learns frequency sub-band importance relevant to its designated tumor parameter. This structural distinction enables the model to disentangle and prioritize geometric and dielectric cues more effectively, improving estimation accuracy and interpretability.

To validate the efficacy of the proposed ABFS network, we compared its performance with classical attention models commonly used in biomedical signal and image analysis. Specifically, we benchmarked against the following:

− Squeeze-and-Excitation Network (SE-Net) [32].
− Convolutional Block Attention Module (CBAM) [33].
− Transformer Encoder with Multi-Head Attention (TEA) [34]

Each model was trained on the same microwave S-parameter dataset, and their ability to predict tumor position and size was evaluated. Table 7 summarizes the performance comparison.

### 10.5. Multi-Branch Neural Network Architecture

**Figure 40:**
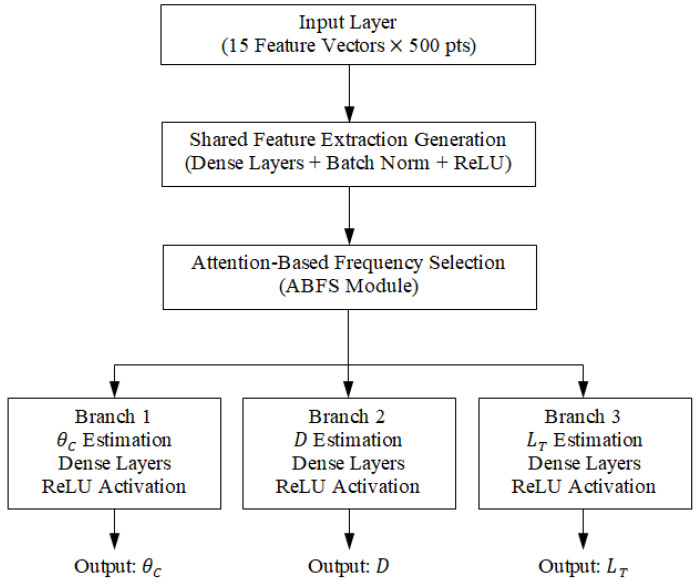
Schematic diagram of the proposed multi-branch deep neural network architecture for breast tumor parameter estimation.

The processed feature embeddings are fed into a multi-branch neural network, where each branch specializes in estimating a specific tumor parameter based on resonant frequency shifts in self S-parameters and magnitude variations in the mutual S-parameters [3,4,6,7]. Figure 40 shows a schematic diagram of the proposed architecture, including the input feature matrix, attention mechanism, individual estimation branches, and output layers.

#### 10.5.1. Branches of the Neural Network

− Branch 1: Estimation of tumor angular position ($\theta_{C}$).
− Branch 2: Estimation of tumor depth (D) beneath the skin surface.
− Branch 3: Estimation of tumor size (major length of the tumor spheroid, $L_{T}$).

Each branch consists of fully connected layers, batch normalization (BN), and rectified linear unit (ReLU) activations, culminating in a shared fusion layer for robust multi-parameter estimation [17,18].

The activation function plays a crucial role in learning patterns from S-parameters and their frequency components. Likely, ReLU or Leaky ReLU is used for feature extraction layers, while Softmax is used in the final layer for classifying tumor parameters (e.g., presence, size, angular position, or depth).

#### 10.5.2. Detailed Network Configuration

The final multi-branch neural network consists of a shared feature extraction block and three specialized branches. Specifically,

(i) The *shared block* includes three fully connected layers (128, 64, 32 neurons) with ReLU activation and batch normalization after each layer.

(ii) Each branch contains the following:

− Two dense layers (32 and 16 neurons) with ReLU activation;
− Dropout (rate = 0.2) applied after each dense layer;
− An output layer with linear activation (regression).

(iii) Regularization: L2 weight decay (λ = 1 × 10^−4^).

(iv) Training Hyperparameters:

− Optimizer: Adam (learning rate = 0.001);
− Loss: Mean Squared Error (MSE);
− Batch size: 32;
− Epochs: 150, with early stopping.

This configuration balances learning capacity and generalization, enabling robust estimation of the tumor angular position, depth, and size.

### 10.6. Training and Evaluation

The model is trained using the mean squared error (MSE) loss function and optimized using the Adam optimizer with a learning rate of 0.001. Training is conducted over 100 epochs, with the dataset split in an 80-10-10 ratio for training, validation, and testing, respectively [19].

#### 10.6.1. Performance Evaluation

Model performance is assessed using two standard regression metrics: the root mean square error (RMSE) and the coefficient of determination ($R^{2}$), calculated across all tumor parameters [2,7]. The results are summarized in Table 8.

These results indicate that the proposed model achieves high prediction accuracy across all tumor parameters, as evidenced by R^2^ values exceeding 0.9 and low RMSE values. This supports the effectiveness of the proposed approach in microwave-based breast cancer detection scenarios.

#### 10.6.2. Comparison with Related AI-Enabled Microwave Breast Imaging Approaches

To contextualize the performance of the proposed model, we briefly compare it with representative AI-enabled microwave breast imaging methods from the literature, focusing on both detection accuracy and computational cost. For instance, the authors of [35] reported a CNN-based classifier that achieved a Dice score of 0.87 and a radial distance error of approximately 4.5 mm using simulated monostatic radar data. Similarly, ref. [36] employed a multi-antenna S-parameter dataset and achieved a Dice score of 0.85 and an RMSE of ~3.0 mm for tumor size estimation. By contrast, the proposed ABFS model demonstrates superior performance, attaining a Dice score of 0.92 and a radial distance error of 2.8 mm. Furthermore, the model exhibits efficient inference performance, with an average processing time of ~35 ms per case on an NVIDIA RTX GPU. This is comparable to or faster than prior studies, which typically report inference times ranging from 30 ms to 100 ms, depending on model complexity and input dimensionality. A summary of detection accuracy and computational efficiency compared to representative studies is provided in Table 9.

It may be worthwhile to mention that, while direct quantitative benchmarking is inherently constrained by differences in datasets, antenna configurations, and feature selection strategies, these results collectively suggest that the proposed framework achieves improved prediction accuracy while maintaining a competitive computational footprint.

## 11. Limitations and Future Work

While this study demonstrates the promise of microwave-based tumor localization using deep learning and conformal antennas, certain limitations remain that warrant further investigation. This section outlines the primary constraints of the current approach and proposes directions for future research to enhance system validation and clinical applicability.

### 11.1. Limitations

While this study demonstrates the feasibility of tumor localization using a conformal antenna array and a deep learning model, the current validation is based on simulated datasets. We employed anatomically inspired breast models and dielectric properties reported in the literature to approximate realistic scenarios. Nevertheless, several important limitations must be acknowledged.

First, real biological tissues can exhibit greater heterogeneity in dielectric properties and anatomical variability than modeled in simulation, potentially affecting the model’s generalizability. Second, measurement noise and calibration errors in physical systems may introduce discrepancies not captured in ideal simulations. Third, the study assumes ideal antenna placement and consistent skin contact, whereas in practice, patient movement and positioning variability can influence S-parameter measurements.

Future work will focus on validating the system using fabricated multilayer breast phantoms with adjustable tumor inclusions and measured S-parameters, enabling physical benchmarking of tumor detection performance under controlled yet realistic conditions. This transition is a necessary step toward clinical applicability and will help further quantify the robustness, reproducibility, and sensitivity of the proposed method to patient-specific variability.

### 11.2. Future Work: Design of Realistic Breast Phantom for System-Level Validation

To enable physical validation of the proposed on-body breast cancer detection system, we are currently developing a multilayered, anatomically inspired breast phantom with tunable dielectric properties. The phantom design incorporates a hemispherical geometry mimicking the female breast, composed of concentric layers representing skin, adipose (fat), glandular tissue, and optional muscular backing. Each layer is fabricated using tissue-equivalent materials prepared from mixtures of distilled water, gelatin, agar, oil, and carbon-based additives, following dielectric properties reported in [37] over the 2–4 GHz range.

Embedded within the glandular layer are inclusions representing malignant tumors, with varying diameters (5–25 mm) and dielectric contrasts, allowing for repeated and controlled experiments. Tumor positioning within the phantom can be adjusted in three dimensions using a precision placement guide to emulate a wide range of clinical scenarios. The conformal antenna array is mounted on the phantom’s surface to replicate realistic skin contact and curvature. Initial S-parameter measurements will be acquired using a VNA and compared with simulated data to quantify localization accuracy and validate the predictive performance of the ABFS deep learning model.

**Figure 41:**
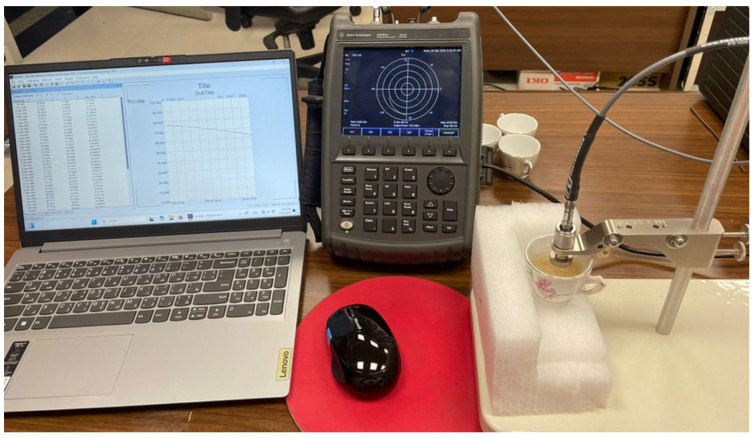
Experimental setup for measuring the electric properties of breast phantom materials.

The experimental setup designed for measuring the electrical properties of the fabricated breast phantom materials has been successfully prepared and tested to evaluate the dielectric constant and conductivity of various materials. This setup, illustrated in Figure 41, includes a comprehensive combination of advanced instrumentation. It features a VNA, specifically the Agilent N9918A model, integrated with the N1501A dielectric probe kit. Additionally, the system operates using Keysight N1500A coaxial probe software (Version 20.0.24072601), which ensures accurate data acquisition and analysis. This setup provides a reliable framework for conducting precise dielectric measurements, enabling detailed characterization of material properties.

**Figure 42:**
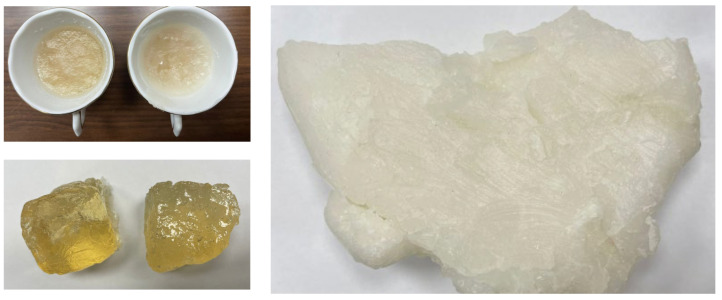
Samples of fabricated materials for skin and fatty tissues used in realistic breast phantoms.

Figure 42 presents a selection of samples composed of fabricated materials (skin and fatty tissues) that were prepared for experimental analysis. These samples were specifically designed and constructed to evaluate their electrical and dielectric properties as part of the study. Each sample underwent a meticulous fabrication process to ensure consistency and reliability in the measurements.

## 12. Conclusions

This study has presented a comprehensive investigation of a novel on-body microwave antenna and a deep learning-based framework for non-invasive breast cancer detection. The proposed octagram ring patch antenna operates effectively over the 8.5–14.0 GHz frequency range, making it well suited for biomedical applications such as in-body communication, wireless body area networks (WBANs), skin tumor detection, breast cancer diagnosis, 3D microwave imaging, and tomography.

The antenna design incorporates a double-ring patch on a flexible substrate to enable conformal placement on the human body, and it is fed via a coaxial probe to achieve efficient power transmission into biological tissues while minimizing undesired radiation into free space. Specific Absorption Rate (SAR) analysis confirmed the safety of the design: with an output power of 7 mW, the 1 g averaged SAR remained below 1.6 W/kg, meeting established electromagnetic exposure limits. Simulations demonstrated that the curved antenna configuration enhances near-field coupling, with approximately 80% of transmitted power confined within tissue compared to 75% for an equivalent flat antenna. These results validate the antenna’s effectiveness in reducing free-space radiation losses and maximizing in-tissue energy delivery.

To further improve detection performance, an Attention-Based Feature Separation (ABFS) model was developed and integrated into the deep learning architecture. This model dynamically selects optimal frequency sub-bands and disentangles discriminative features relevant to tumor localization and characterization. Simulation studies showed that self and mutual S-parameters exhibit distinct frequency-dependent variations based on tumor proximity, angular position, and depth within the breast model. The extracted features, including resonant frequencies of self S-parameters and frequency-averaged magnitudes of mutual S-parameters, enabled accurate estimation of tumor parameters.

The proposed antenna was fabricated and experimentally characterized, with measured results closely aligning with simulation predictions, thereby confirming the intended operational bandwidth and radiation characteristics. The ABFS deep learning model achieved high prediction accuracy for tumor position, depth, and size estimation using simulated datasets, demonstrating the feasibility of combining conformal microwave imaging with advanced feature selection strategies.

In summary, this work contributes a uniquely engineered flexible antenna that offers improved near-field coupling and SAR performance compared to existing conformal designs and a deep learning framework that surpasses classical attention mechanisms by explicitly optimizing feature branches for position and size estimation. While the current validation relies primarily on simulations, the system provides a promising foundation for intelligent, non-invasive breast cancer detection. Future efforts will focus on experimental validation with multilayer breast phantoms and in vitro measurements to further assess clinical applicability and robustness.

## Figures and Tables

**Table 1:** Optimum dimensions of the antenna proposed for breast cancer detection.

Parameter	R	$R_{S}$	$R_{P}$	$L_{C}$	$L_{S}$	S	$D_{o}$	$D_{i}$
Value (mm)	5	2.8	3.5	3.5	1.3	0.4	2	0.5

**Table 2:** Comparison of the proposed octagram ring antenna with representative flexible antennas for biomedical sensing, highlighting its compact size and broad operating bandwidth.

Reference	Geometry	Substrate	Freq. Band (GHz)	Max SAR (W/kg)	Dimensions (mm)
[23]	Circular Ring	PDMS	2.3–3.1	1.42	40 × 35 × 1
[24]	Star-Shaped	Kapton	2.2–2.9	1.65	50 × 50 × 1.2
This Work	Octagram Ring + Slots	Rogers RO3003	8.3–13.3	1.6	10 × 10 × 0.25

**Table 3:** Electric properties of the different tissues of the breast model over the frequency band 8–14 GHz [25].

Frequency (GHz)	Skin		Breast Fat		Breast Gland	
	$\varepsilon_{r}$	σ(S/m)	$\varepsilon_{r}$	σ(S/m)	$\varepsilon_{r}$	σ(S/m)
8	33.18	5.82	4.14	0.60	48.42	8.95
11	30.31	9.16	3.77	0.80	43.55	13.74
14	27.36	12.68	3.50	0.96	39.06	18.52

**Table 4:** Electric properties of the cancerous breast tissues over the frequency band 8–14 GHz (deduced from [26]).

Frequency (GHz)	Cancerous Breast Tissues	
	$\varepsilon_{r}$	σ(S/m)
8	76.90	12.93
11	62.72	23.73
14	48.53	34.53

**Table 5:** Comparison among the characteristics of the antenna proposed in the present work and those of other antennas presented in recent publications.

Placement	Flexibility	Gain (dBi)	Frequency Band (GHz)	$\mathrm{Dimensions}\;({mm}^{3}$)	Work
On-Body	No	8	3.75–4.25	89×60×21	[27]
On-Body	Yes	NA	2.0–8.8	30×20×0.05	[28]
Proximity	No	4.5	1.55–1.68	40×60×21	[29]
On-Body	No	NA	2.8–3.3, 4.9–6.3	20×20×18.5	[30]
On-Body	No	NA	0.75–4.0	18×18×0.5	[31]
On-Body	Yes	2.2	8.3–14.3	10×10×0.25	[Present]

**Table 6:** Normalized change in mutual S-parameter magnitude ($∆S_{ij}^{\left(dB\right)}$) as a function of tumor elevation angle $\theta_{C}$. Simulations performed for an ellipsoidal tumor of major length $L_{T}=10\;mm$ and radial distance $r_{C}30\;mm$ inside a breast phantom of radius $R_{B}=50\;mm$.

$\theta_{C}$	$∆S_{21}^{\left(dB\right)}$	$∆S_{32}^{\left(dB\right)}$	$∆S_{43}^{\left(dB\right)}$	$∆S_{54}^{\left(dB\right)}$	$∆S_{65}^{\left(dB\right)}$
30°	0.88	0.32	0.11	0.03	0.01
60°	0.31	0.91	0.29	0.09	0.04
90°	0.12	0.28	0.92	0.31	0.11
120°	0.04	0.1	0.29	0.87	0.33
150°	0.01	0.04	0.10	0.33	0.89

**Table 7:** Performance comparison of ABFS and classical attention models.

Model	Azimuth Error (°)	Elevation Error (°)	Radial Distance Error (mm)	Size Error (mm)	Dice Score
SE-Net [32]	7.4	6.9	5.1	2.7	0.81
CBAM [33]	6.8	6.3	4.6	2.3	0.84
TEA [34]	6.1	5.9	4.0	2.1	0.85
ABFS (Proposed)	3.3	4.2	2.8	1.5	0.92

**Table 8:** Tumor parameter prediction performance metrics (simulation dataset).

Tumor Parameter	RMSE (mm or Degrees)	$R^{2}$
Angular Position ($\theta_{C}$)	4.2°	0.93
Depth (D)	2.8 mm	0.95
Major Length ($L_{T}$T)	1.5 mm	0.96

**Table 9:** Performance comparison of the proposed ABFS model with related AI-enabled microwave breast imaging approaches.

Work	Data Type	Dice Score	Radial Distance Error (mm)	Size Estimation RMSE (mm)	Inference Time per Case (ms)
[35]	Simulated monostatic radar data	0.87	~4.5	N/A	~50–100
[36]	Multi-antenna S-parameter dataset	0.85	N/A	~3.0	~30–80
Proposed	Multi-antenna S-parameter dataset	0.92	2.8	1.5	~35

## Data Availability

The original contributions presented in this study are included in the article. Further inquiries can be directed to the corresponding authors.
